# Activating Transcription Factor 5 Promotes Neuroblastoma Metastasis by Inducing Anoikis Resistance

**DOI:** 10.1158/2767-9764.CRC-23-0154

**Published:** 2023-12-12

**Authors:** Debarshi Banerjee, Shuobo Boboila, Shunpei Okochi, James M. Angelastro, Angela V. Kadenhe-Chiweshe, Gonzalo Lopez, Andrea Califano, Eileen P. Connolly, Lloyd A. Greene, Darrell J. Yamashiro

**Affiliations:** 1 Department of Pediatrics, Columbia University Irving Medical Center, New York, New York.; 2 Department of Radiation Oncology, Columbia University Irving Medical Center, New York, New York.; 3 Department of Surgery, Columbia University Irving Medical Center, New York, New York.; 4 Department of Molecular Biosciences, University of California, Davis, School of Veterinary Medicine, Davis, California.; 5 Department of Surgery, Weill Cornell Medicine, New York, New York.; 6 Department of Systems Biology, Columbia University Irving Medical Center, New York, New York.; 7 Herbert Irving Comprehensive Cancer Center, Columbia University Irving Medical Center, New York, New York.; 8 Department of Pathology and Cell Biology, Columbia University Irving Medical Center, New York, New York.

## Abstract

*MYCN*-amplified neuroblastoma often presents as a highly aggressive metastatic disease with a poor prognosis. Activating transcription factor 5 (ATF5) is implicated in neural cell differentiation and cancer cell survival. Here, we show that ATF5 is highly expressed in patients with stage 4 high-risk neuroblastoma, with increased expression correlating with a poorer prognosis. We demonstrated that ATF5 promotes the metastasis of neuroblastoma cell lines *in vivo*. Functionally, ATF5 depletion significantly reduced xenograft tumor growth and metastasis of neuroblastoma cells to the bone marrow and liver. Mechanistically, ATF5 endows tumor cells with resistance to anoikis, thereby increasing their survival in systemic circulation and facilitating metastasis. We identified the proapoptotic BCL-2 modifying factor (BMF) as a critical player in ATF5-regulated neuroblastoma anoikis. ATF5 suppresses BMF under suspension conditions at the transcriptional level, promoting anoikis resistance, whereas BMF knockdown significantly prevents ATF5 depletion–induced anoikis. Therapeutically, we showed that a cell-penetrating dominant-negative ATF5 peptide, CP-d/n-ATF5, inhibits neuroblastoma metastasis to the bone marrow and liver by inducing anoikis sensitivity in circulating tumor cells. Our study identified ATF5 as a metastasis promoter and CP-d/n-ATF5 as a potential antimetastatic therapeutic agent for neuroblastoma.

**Significance:**

This study shows that resistance to anoikis in neuroblastoma is mediated by ATF5 and offers a rationale for targeting ATF5 to treat metastatic neuroblastoma.

## Introduction

High-risk neuroblastoma is characterized by metastatic spread to the bone, bone marrow, or liver in patients who are greater than 18 months of age or whose tumors have amplification of the proto-oncogene *MYCN* (1, 2). Despite advances in treatment, the overall prognosis of patients with high-risk neuroblastoma remains poor, with a 5-year survival rate of 50% (3, 4). New therapies are urgently needed and will require the identification of novel targets and an understanding of their role in metastasis.

Resistance to anoikis, an apoptotic cell death induced by insufficient cell–matrix interactions, is a critical step in metastasis (5, 6). Anoikis resistance allows tumor cells to survive in the systemic circulation and distant organs, facilitating metastasis. Aberrant activation of prosurvival pathways, deregulated metabolism, and altered cell-matrix signaling are significant contributors to anoikis resistance (7–9). Consequently, anoikis induction in metastatic cancer cells is central to developing antimetastatic therapies.

Activating transcription factor 5 (ATF5), an ATF/CREB family member, is a basic leucine zipper (bZIP) transcription factor. ATF5 is highly expressed in neural progenitor/neural stem cells, where it promotes cell proliferation and inhibits neurogenesis and gliogenesis (10, 11). ATF5 also acts as a prosurvival factor that supports cellular adaptation to stress (10, 12–15) and is highly expressed in multiple malignancies, including glioblastoma, breast cancer, colon cancer, and osteosarcoma (10, 16–18).

We report that ATF5 increases tumor cell survival in circulation by inducing anoikis resistance *in vitro* and *in vivo*, thereby facilitating metastasis. Mechanistically, ATF5 induces anoikis resistance by suppressing the proapoptotic BCL-2 modifying factor (BMF). We also demonstrated that targeting ATF5 using a cell-penetrating dominant-negative ATF5 peptide inhibitor (CP-d/n-ATF5) increased the anoikis sensitivity of tumor cells and inhibited metastasis.

## Materials and Methods

### Cell Culture

Human neuroblastoma cell lines were purchased from the ATCC: BE(2)-C (ATCC, catalog no. CRL-2268, RRID:CVCL_0529), SK-N-DZ (ATCC, catalog no. CRL-2149, RRID: CVCL_1701), and SH-SY5Y (ATCC, catalog no. CRL-2266, RRID: CVCL_0019). Cell lines were authenticated by short tandem repeat profiling (ATCC). *Mycoplasma* testing was performed at the initiation of the project. Cell lines were cultured in DMEM (Thermo Fisher Scientific) supplemented with 10% FBS (Thermo Fisher Scientific).

### Reagents and Plasmids

CP-d/n-ATF5 and penetratin peptides were purchased from CS Bio and were formulated in water as described previously (16). The full-length human open reading frame (ORF) BMF cDNA (NM_001003940.1) cloned into the pCMV3 vector was obtained from Sino Biological. The pCCL-ATF5 expression plasmid was constructed by cloning the human ATF5 (NM_012068) ORF cDNA into the pCCL vector (19). As described previously, plasmid transfection was mediated by FuGENE HD Transfection Reagent (Promega; ref. 20).

### Short Hairpin RNA and siRNA Transfection

Lentivirus-mediated stable integration of the doxycycline (Dox)-inducible pTRIPZ vector (Dharmacon) encoding shATF5-1 or shATF5-2 was performed as described previously (20). shATF5 expression was induced by adding Dox (1 µg/mL). ON-TARGETplus human FOXO3 siRNAs (50 nmol/L) and ON-TARGETplus human BMF siRNAs (50 nmol/L; Dharmacon) were transiently transfected in adherent cells using the DharmaFECT1 reagent (Dharmacon; ref. 20).

### Cell Viability Assay

Cell viability was measured using the CCK-8 assay kit (Dojindo Molecular Technologies; ref. 21). Cells were plated on a 96-well plate at 10,000 cells/well in complete media. At the time of measurement after treatment, 10 µL of Cell Counting Kit-8 solution in each well was added, incubated for 2 hours at 37°C, and absorbance was measured at 450 nm on a microplate reader (Molecular Devices).

### Anoikis Assay

Suspension conditions were created by coating 96-well plates with poly-HEMA (20 mg/mL in 95% ethanol; ref. 22). Cells were seeded on a 96-well plate at 10,000 cells/well in complete media. Anoikis cell death was measured by evaluating DNA fragmentation by terminal deoxynucleotidyl transferase dUTP nick end labeling (TUNEL) assay using the HT TiterTACSApoptosis Detection Kit (R&D Systems), as described previously (21, 23, 24). Briefly, suspension cells were collected by centrifuging the plate at 1,000 × *g* for 3 minutes at room temperature. Cells were fixed with 3.7% formaldehyde for 7 minutes at room temperature, centrifuged, washed with 1X PBS, and post-fixed in 100% methanol for 20 minutes. Cells were treated with Proteinase K Solution for 15 minutes, washed, and treated with 3% hydrogen peroxide to quench endogenous peroxidase. According to the instruction manual, cells were labeled with 1X TdT Labeling Reaction Mix at 37°C for 1 hour, and the reaction was stopped with 1X TdT Stop Buffer for 5 minutes. After washing, cells were mixed with Strep-HRP (horseradish peroxidase) solution for 10 minutes, washed, TACS-Sapphire substrate was added for 30 minutes, and the reaction was stopped with 0.2 mol/L HCl. Cell death was measured absorbance at 450 nm on a microplate reader (Molecular Devices).

### Immunoblot Analysis

Suspension cells were collected by centrifuging at 1,000 × *g* for 3 minutes at room temperature. Cells were washed two times with ice-cold 1X PBS. Cell lysates were made in ice-cold 1X RIPA buffer (MilliporeSigma) containing 1 mmol/L phenylmethylsulfonyl fluoride (MilliporeSigma) and protease inhibitor cocktail (MilliporeSigma; refs. 14, 19). Cell lysates were sonicated for 10 seconds (three times), and the protein concentration of each sample was measured using the Bio-Rad Protein Assay.

Immunoblotting was performed as described previously (25, 26). For antibodies, see Supplementary Table S1. Densitometric analysis was performed using ImageJ (NIH, Bethesda, MD; http://imagej.nih.gov/ij).

### RT-PCR

BE(2)-C and SK-N-DZ cells, expressing shATF5-1 or shATF5-2, were seeded on poly-HEMA–coated 6-well plates in complete media for 24 hours. Dox (1 µg/mL) was added, and 72 hours later, total RNA was purified using an RNeasy Mini Kit (Qiagen) according to the supplier's instruction manual. cDNA was generated by performing RT-PCR using SuperScript III First Strand RT-PCR kit (Invitrogen) according to the manufacturer's protocol. The cDNAs generated were subjected to PCR with human ATF5, BMF, or ACTB cDNA-specific primers.

### Mouse Models

The Columbia University Institutional Animal Care and Use Committee approved all animal procedures. Mice experiments were performed in 6–8 weeks old female NCr nude mice (Taconic). BE(2)-C and SK-N-DZ cells were stably transfected with firefly *luciferase* (19–21, 27). Mice were anesthetized with ketamine and xylazine, an incision was made at the left flank, and 10^6^ cells were injected into the renal parenchyma to establish xenograft tumors. Ten days after implantation mice bearing BE(2)-C tumors were randomized into two groups. One group was given drinking water containing Dox (2 mg/mL) to induce ATF5 knockdown (20), and the other group received drinking water without Dox. Tumor growth was monitored twice per week by bioluminescence imaging (19, 20). For bioluminescence analysis, mice were injected intraperitoneally with 75 mg/kg d-Luciferin (PerkinElmer), anesthetized with isoflurane, and imaged with a Xenogen IVIS200 (19, 20). Mice were euthanized when bioluminescence flux reached a threshold of 4 × 10^9^ (photons/second).

For CP-d/n-ATF5 treatment, kidney xenograft tumors, mice were treated with 50 mg/kg of the peptide, or vehicle, by intraperitoneal injection once per day for the first 3 days and then twice per week (16). For BE(2)-C tumors, treatment was started 7 days after cell implantation. Tumor growth was monitored by bioluminescence, and mice were euthanized when bioluminescence reached a threshold flux (4 × 10^9^ photons/second). For SK-N-DZ tumors, peptide treatment began 14 days after cell implantation. Mice were euthanized 40 days (day 40) after cell implantation.

### Intracardiac Injection

A total of 10^6^ cells were injected into the left cardiac ventricle of nude mice under isoflurane inhalation. Mice were given drinking water with Dox (2 mg/mL) or without Dox 3 days before injection to the time of euthanasia. Bioluminescence imaging was obtained 20 minutes after injection to monitor the distribution of tumor cells. Twelve hours after intracardiac injection, mice were euthanized and blood was collected. A cohort of mice was also euthanized 31 days (day 31) after cell injection.

For peptide inhibitor studies, CP-d/n-ATF5, at a dose of 50 mg/kg, was intraperitoneally injected into mice 1 hour after intracardiac injection of tumor cells, and mice were euthanized 12 hours later or 38 days (day 38) after cell injection.

### Circulating Tumor Cells

Blood was collected from the submandibular vein of mice and lysed by centrifugation at 10,000 × *g* for 30 minutes. The supernatant was mixed with LARII reagent (Promega), and bioluminescence was measured using a luminometer (19).

### Isolation of Circulating Tumor Cells

At the time of euthanasia, blood was collected, diluted with PBS + 2% FBS, and layered on top of Lymphoprep (STEMCELL Technologies). After centrifugation at 800 × *g* for 20 minutes, the mononuclear cell layer was removed, washed, resuspended in buffer (PBS + 0.5% BSA, pH 7.2), and incubated with an anti-GD2-FITC antibody for 1 hour at 4°C. The cells were washed twice and incubated with FITC microbeads (Miltenyi Biotec) for 30 minutes at 4°C. The cells were washed, resuspended, and applied to an MS column placed in a MACS separator (Miltenyi Biotec). The labeled cells were flushed out and counted with 1X trypan blue, and equal numbers of cells were subjected to the TUNEL assay to determine cell death or mRNA expression of *ATF5*, *BMF,* and *ACTB* by RT-PCR.

### Assessment of Liver and Bone Marrow Metastasis

For *ex vivo* liver imaging, mice were injected with d-luciferin, euthanized, and livers were dissected, imaged, and bioluminescence measured (19). Bone marrow metastases were evaluated by flushing cells from the ends of isolated femurs, centrifuging, and lysing the cells in passive lysis buffer (Promega). The supernatant was mixed with LARII reagent (Promega), and bioluminescence was measured using a luminometer and normalized to the number of cells (19).

### Statistical Analysis

Statistical analyses were performed using Prism software (GraphPad). Survival was determined using the log-rank test (Mantel–Cox). Data were analyzed using an unpaired *t* test. Bioluminescence data were transformed to *Y* = log(*Y*).

### Data Availability

The data from the Sequencing Quality Control Consortium (SEQC) cohort used in this study can be obtained from Gene Expression Omnibus (GEO): GSE4971 (28). The tumor genomics data from the TARGET cohort (29) are available through the data matrix portal (https://ocg.cancer.gov/programs/target/data-matrix). All other data in support of the findings can be obtained from the Corresponding Author upon reasonable request.

## Results

### ATF5 is Expressed in High-risk Neuroblastoma Tumors

**FIGURE 1:**
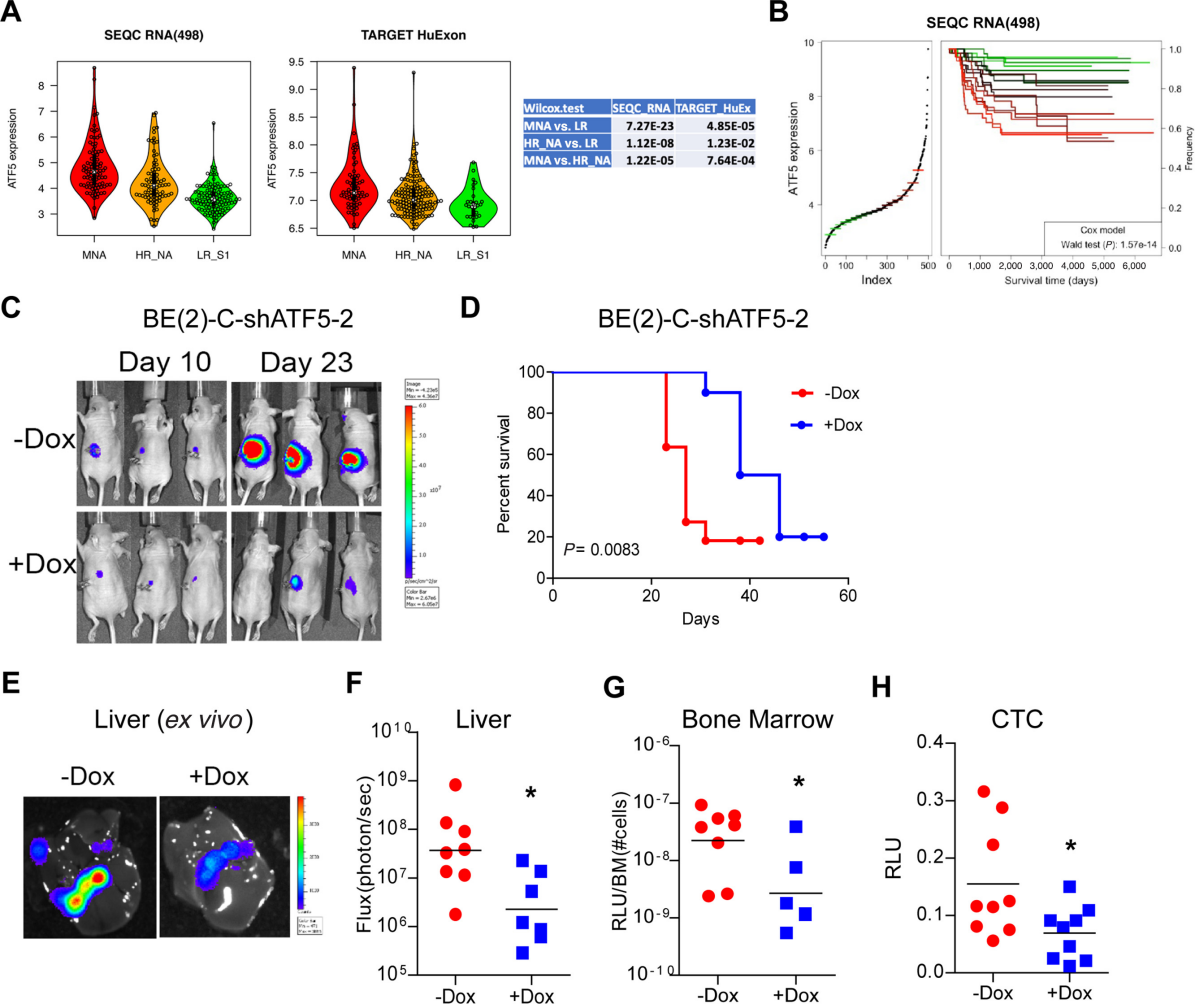
Elevated *ATF5* expression correlates with poor outcomes in patients with neuroblastoma, and ATF5 silencing prolongs survival and inhibits metastatic progression in neuroblastoma-bearing mice. **A,** Violin plots of *ATF5* expression in high-risk *MYCN-*amplified (MNA, red), high-risk *MYCN*-non-amplified (HR_NA, yellow), and low-risk stage 1 (LR_S1, green) patients. For SEQC, MNA = 92, HR_NA = 83, LR_S1 = 118. For TARGET, MNA = 68, HR_NA = 145, LR_S1 = 30. Expression data: SEQC (from GEO: GSE49711): https://www.ncbi.nlm.nih.gov/geo/query/acc.cgi?acc=GSE49711; ref. 28); TARGET: HumanExon arrays (29). **B,** Kaplan–Meier curve depicting the corresponding increase in poor outcome with increasing *ATF5* expression. The *P* value was calculated using a Cox proportional hazards model after removing stage 1 patient samples, Wald test, *P* = 1.57E-14. **C,** Bioluminescent images at day 10 and day 23 of mice bearing Dox-inducible BE(2)-C-shATF5-2 renal xenograft tumors and treated with or without Dox. **D,** Kaplan–Meier plot of mice bearing Dox-inducible BE(2)-C-shATF5-2 renal xenograft tumors with ATF5 knocked down (+ Dox, *n* = 10) or not (− Dox, *n* = 11). *P* = 0.0083, log-rank (Mantel–Cox). **E,***Ex vivo* bioluminescent images of the liver at the time of euthanasia**. F,** Quantification of total flux (photons/second) by *ex vivo* liver bioluminescence at the time of euthanasia. **G,** Quantification of bone marrow metastases from bioluminescence of bone marrow homogenate (RLU/# BM cells) at the time of euthanasia. RLU: relative luciferase unit. **H,** Quantification of bioluminescence from blood representing the number of CTCs at the time of euthanasia. *, *P* < 0.05.

Examining neuroblastoma patient datasets from the SEQC cohort (GEO: GSE49711; ref. 28) and the NCI TARGET cohort (29), we found that *ATF5* expression (Fig. 1A) was significantly higher in *MYCN*-amplified (MNA) tumors than in either high-risk *MYCN*-non-amplified (HR_NA) tumors (SEQC cohort *P =* 1.22E-05; TARGET cohort *P =* 7.64E-04) or stage 1 low-risk (LR) tumors (SEQC cohort *P*  =  7.27E-23, TARGET cohort *P*  =  4.85E-5). HR_NA tumors also had higher *ATF5* expression than LR tumors (SEQC cohort *P* = 1.12E-08, TARGET cohort *P*  =  1.22E-02). To determine whether *ATF5* expression correlated with patient survival, we examined the SEQC cohort because it is unbiased with respect to clinical factors. Analysis of the SEQC cohort revealed that *ATF5* expression (Fig. 1B) was a strong negative predictor of patient survival (Wald test, *P*  =  1.57E-14).

### ATF5 is Expressed in Neuroblastoma Cell Lines

Immunoblotting revealed that ATF5 was expressed in both *MYCN*-amplified and *MYCN*-non-amplified cell lines (Supplementary Fig. S1A). In line with ATF5 functioning as a transcription factor (14), nuclear ATF5 was detected by immunofluorescence in BE(2)-C and SK-N-DZ cells (Supplementary Fig. S1B).

### ATF5 is Critical for Neuroblastoma Tumor Growth and Metastatic Progression

To investigate the role of ATF5 role in neuroblastoma, we performed loss-of-function studies using two independent Dox-inducible pTRIPZ lentiviral short hairpin RNAs (shRNA) targeting ATF5. ATF5 knockdown by Dox addition reduced cell viability by an average of 40% in BE(2)-C and 50% in SK-N-DZ at 72 hours, compared with controls (Supplementary Fig. S2A). ATF5 knockdown increased apoptosis by an average of 45% in BE(2)-C cells and 50% in SK-N-DZ cells (Supplementary Fig. S2B).

We next investigated the oncogenic function of ATF5 *in vivo.* BE(2)-C-shATF5-2 cells were implanted intrarenally in nude mice. Mice were randomized after 10 days, and half were given drinking water containing Dox to induce ATF5 knockdown*.* Survival time was defined as the day when the primary tumor luciferase flux reached 4 ×  10^9^ photons/second. ATF5 knockdown inhibited tumor growth (Fig. 1C) and significantly increased median survival (Fig. 1D; 42 vs. 27 days, + Dox vs. −Dox, respectively, *P*  =  0.0083). Decreased expression of ATF5 in + Dox tumors was verified by immunoblotting (Supplementary Fig. S3A), indicating that tumor progression was not due to an escape from ATF5 knockdown. Tumors in which ATF5 was knocked down also exhibited increased apoptosis (2.5-fold; Supplementary Fig. S3B).

The effect of ATF5 knockdown on liver and bone marrow metastasis and circulating tumor cells (CTC) was also examined. ATF5 knockdown markedly decreased the liver metastatic burden, with a 22-fold lower total liver flux than controls (Fig. 1E and F). ATF5 knockdown also decreased bone marrow metastasis, with a 4.5-fold decrease in bioluminescence in bone marrow cell homogenates (Fig. 1G). ATF5 knockdown also decreased CTCs in the blood, with a 2-fold reduction in bioluminescence (Fig. 1H). These data demonstrate a role for ATF5 in promoting metastasis to the bone marrow and liver and in increasing the number of CTCs.

### Overexpression of ATF5 Enhances Anoikis Resistance

**FIGURE 2:**
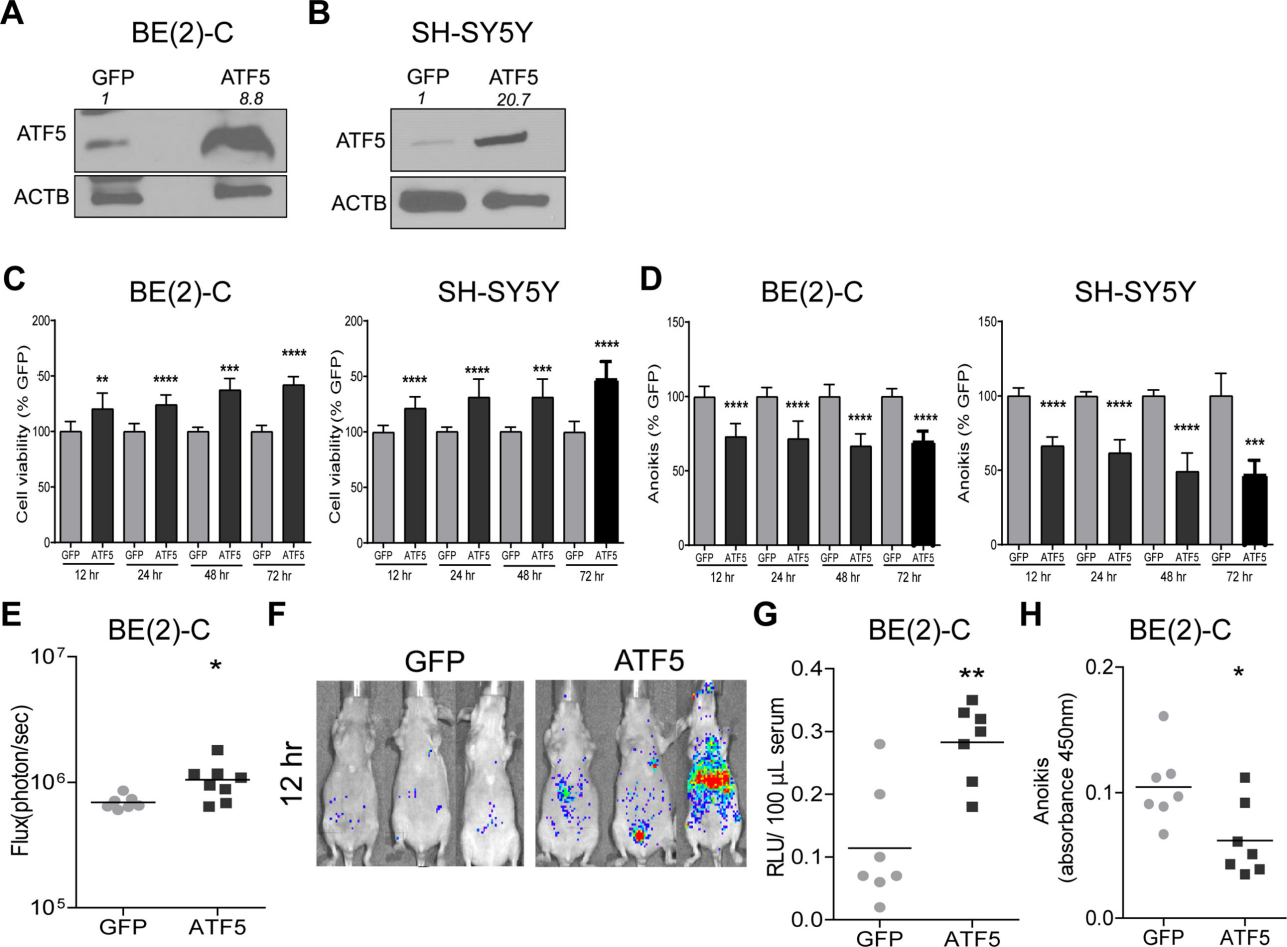
Overexpression of ATF5 promotes anoikis resistance *in vitro* and *in vivo*. **A** and **B,** ATF5 overexpression, by immunoblot, in suspension culture of BE(2)-C and SH-SY5Y cells, 72 hours after transient transfection with pCCL-GFP or pCCL-ATF5. ACTB was used as a loading control. Densitometric analysis was performed using ImageJ. **C** and **D,** BE(2)-C and SH-SY5Y suspension cell viability and anoikis at 12, 24, 48, and 72 hours after transfection, respectively. At each timepoint, the comparison is between cells overexpressing ATF5 and control cells expressing GFP (100%). Mean ± SD. **E,** Quantification of mouse whole-body bioluminescence flux (photons/second) 12 hours after intracardiac injection of BE(2)-C cells overexpressing ATF5 (*n* = 8) or GFP (*n* = 7). **F,** Images showing increased bioluminescence of animals described in E indicate tumor cell survival 12 hours after intracardiac injection. **G,** Quantification of bioluminescence of blood collected 12 hours after intracardiac injection as in E. **H,** Quantification of apoptosis of BE(2)-C CTC isolated at 12 hours, GFP (*n* = 7); ATF5 (*n* = 7). *, *P* < 0.05; **, *P* < 0.01; ***, *P* < 0.001; ****, *P* < 0.0001.

Next, we investigated the mechanisms underlying the decreased CTC levels and metastasis seen with ATF5 knockdown. ATF5 knockdown did not influence the invasiveness of BE(2)-C cells utilizing an extracellular matrix basement membrane assay (Supplementary Fig. S4). We then evaluated the role of ATF5 in tumor cell anchorage-independent viability and anoikis resistance, which are critical prerequisites for metastasis. To determine whether overexpression of ATF5 can increase resistance to anoikis, we transiently overexpressed ATF5 in BE(2)-C cells (Fig. 2A) and in the *MYCN*-non-amplified cell lines SH-SY5Y (Fig. 2B), and CHLA-255 (Supplementary Fig. S5A). Overexpression of ATF5 increased anchorage-independent viability of BE(2)-C (Fig. 2C), SH-SY5Y (Fig. 2C), and CHLA-255 cells (Supplementary Fig. S5B). In addition, forced ATF5 expression decreased anoikis of BE(2)-C (Fig. 2D), SH-SY5Y (Fig. 2D), and CHLA-255 cells (Supplementary Fig. S5C).

We also examined the effect of ATF5 overexpression on tumor cell survival *in vivo*. Twelve hours after intracardiac injection of ATF5-overexpressing BE(2)-C cells, mice exhibited a marked elevation in whole-body flux (Fig. 2E and F) compared with mice with GFP-expressing cells. ATF5 overexpression also elevated the number of CTC (2.5-fold) compared with GFP-expressing CTC (Fig. 2G). Isolation of CTC and performing a TUNEL assay demonstrated a 2-fold reduction in apoptosis in CTC overexpressing ATF5 compared with CTCs expressing GFP (Fig. 2H, *P* < 0.05).

### ATF5 is Required for Resistance to Anoikis

**FIGURE 3:**
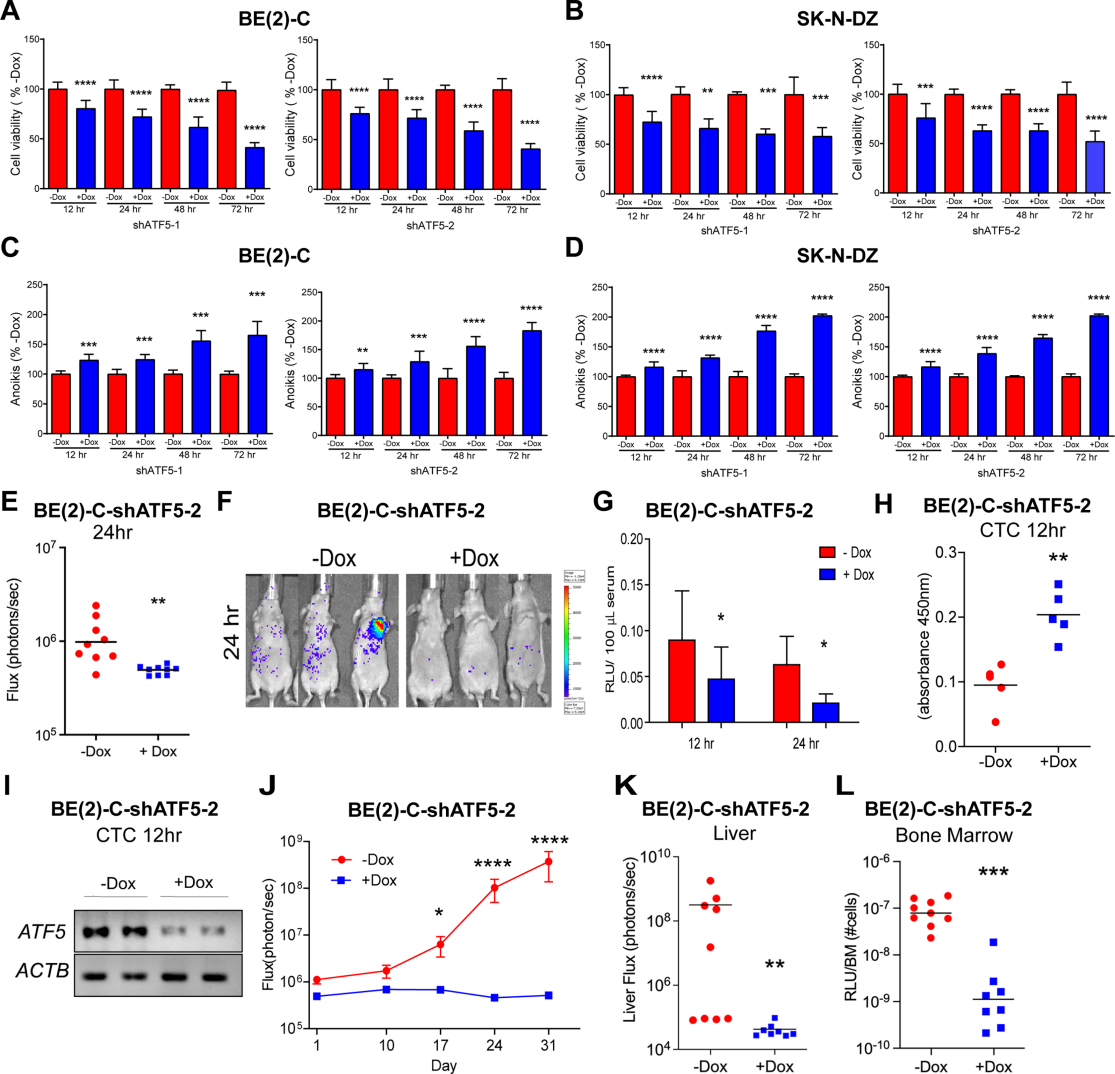
Depletion of ATF5 induces anoikis and decreases metastasis of neuroblastoma cells. **A** and **B,** Viability of BE(2)-C and SK-N-DZ suspension cells in poly-HEMA–coated plates, expressing Dox-inducible shATF5-1 or shATF5-2 at different timepoints after Dox treatment. Mean ± SD. **C** and **D,** Quantification of anoikis of BE(2)-C and SK-N-DZ suspension cells cultured as in A and B at different timepoints after Dox addition. Mean ± SD. **E,** Quantification of whole-body bioluminescence flux (photons/second) in mice 24 hours after intracardiac injection of BE(2)-C-shATF5-2 cells, + Dox (*n* = 9); −Dox, (*n* = 9). Mice were maintained on drinking water containing Dox (2 mg/mL) from 3 days before injection to the time of euthanasia. **F,** Bioluminescent images at 24 hours after intracardiac injection under conditions described in E. **G,** Quantification of bioluminescence of blood from mice collected 12 and 24 hours after intracardiac injection under conditions described in E. **H,** Quantification of apoptosis of BE(2)-C-shATF5-2 CTCs isolated from mice 12 hours after intracardiac injection and treatment ± Dox (see E and Materials and Methods), + Dox (*n* = 5), −Dox, (*n* = 5). **I,** RT-PCR analyses of *ATF5* and *ACTB* in circulating BE(2)-C-shATF5-2 cells isolated after 12 hours. **J,** Time course of whole-body bioluminescence flux in mice treated as in E. + Dox (*n* = 8); −Dox, (*n* = 9). The mice were monitored for metastatic spread by bioluminescence and euthanized at day 31. **K,** Quantification of total flux (photons/second) by *ex vivo* liver bioluminescence at the time of euthanasia (day 31). **L,** Quantification of bioluminescence in bone marrow homogenates at the time of euthanasia (day 31). *, *P* < 0.05; **, *P* < 0.01; ***, *P* < 0.001; ****, *P* < 0.0001.

We next investigated the effects of ATF5 knockdown on resistance to anoikis. Anoikis was induced by culturing the cells in suspension in poly-HEMA–coated plates. A time-course study with two different Dox-inducible shRNAs showed that ATF5 knockdown in BE(2)-C cells caused a decrease in anchorage-independent viability by 22% at 12 hours and 59% at 72 hours (Fig. 3A). An accompanying increase in apoptosis was observed, with a rise of 19% at 12 hours and 74% at 72 hours (Fig. 3C). Similar results were found in SK-N-DZ cells, with ATF5 knockdown decreasing anchorage-independent viability (Fig. 3B) and increasing apoptosis (Fig. 3D). Immunoblot analyses verified a time-dependent decrease in ATF5 after Dox addition (Supplementary Fig. S6). These findings suggest that ATF5 promotes resistance to anoikis and anchorage-independent growth of neuroblastoma cells and that its loss decreases their anchorage-independent viability.

### ATF5 Promotes Metastasis by Suppressing CTC Anoikis

Increased anoikis upon ATF5 knockdown encouraged us to hypothesize that ATF5 promotes the survival of CTCs, thereby facilitating metastasis. To test this, *luciferase-*expressing BE(2)-C-shATF5 cells were injected intracardially into nude mice fed with or without Dox-containing water. A significant decrease in whole-body flux was noted at 24 hours in +Dox-treated mice compared with that in −Dox control mice (Fig. 3E and F). Quantification of CTCs demonstrated a 2-fold decrease at 12 hours and a 3-fold reduction at 24 hours in + Dox mice compared with −Dox control mice (Fig. 3G). CTCs were isolated 12 hours after intracardiac injection and analyzed by TUNEL assay for apoptosis to determine whether the decrease in CTC was due to increased apoptosis. There was a 2-fold increase in CTC apoptosis (Fig. 3H) in the +Dox group compared with −Dox. Increased CTC apoptosis was associated with reduced *ATF5* mRNA levels (Fig. 3I). To demonstrate that the effect of *ATF5* knockdown on CTC survival was not cell line–specific, we intracardially injected SK-N-DZ-shATF5 cells into mice pretreated with Dox. Mice receiving Dox showed a significant decrease in whole-body flux (Supplementary Fig. S7A and S7B) and a 2-fold increase in CTC apoptosis at 12 hours compared with controls (Supplementary Fig. S7C).

To determine whether ATF5 knockdown could impact metastasis, we intracardiac injected BE(2)-C-shATF5 cells and followed whole-body bioluminescence flux over time (Fig 3J). In the +Dox-treated mice, there was no increase in whole-body flux, in contrast to the increase in flux seen in −Dox mice. On day 31, mice were euthanized, and metastasis was evaluated. ATF5 knockdown resulted in a 5,000-fold decrease in liver flux (Fig. 3K) and bone marrow homogenate bioluminescence (Fig. 3L). These data indicate that ATF5 knockdown induces apoptosis of CTCs, decreasing their survival in circulation, thereby markedly reducing liver and bone marrow metastases.

### Knockdown of ATF5 Induces BMF

**FIGURE 4:**
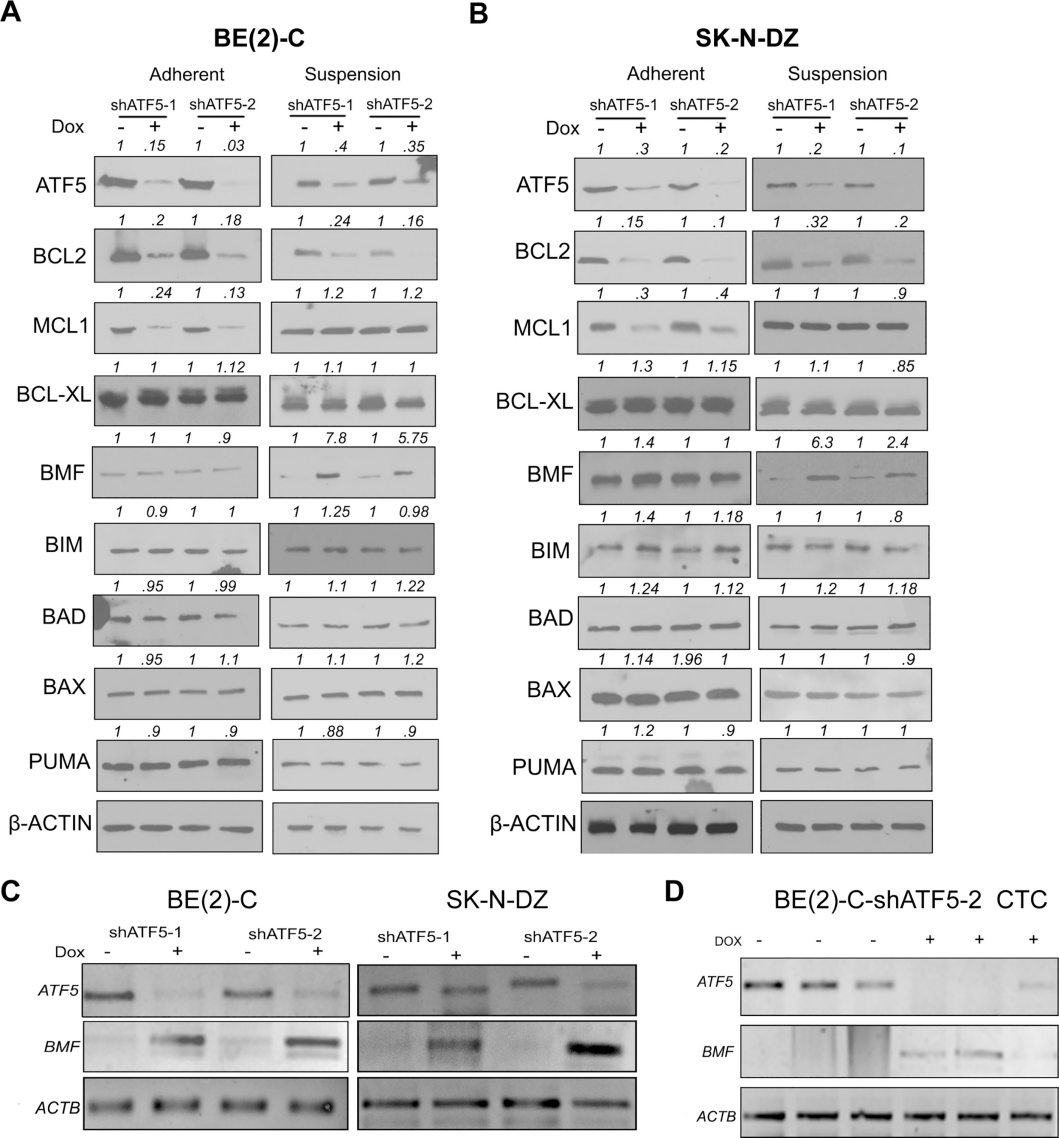
ATF5 depletion induces BMF in suspension culture. Immunoblot of BCL-2 family proteins in BE(2)-C cells (**A**) and SK-N-DZ cells (**B**) expressing Dox-inducible shATF5-1 or shATF5-2, in adherent and suspension cultures, respectively, 72 hours after treatment ± Dox. β-Actin was used as a loading control. Densitometric analysis was performed using ImageJ. **C,** RT-PCR analyses of *ATF5* and *BMF* in detached BE(2)-C cells 72 hours after ±Dox addition. RNA was isolated, reverse transcribed, and PCR was performed. *ACTB* was used as an internal control. **D,** RT-PCR of *ATF5* and *BMF* in circulating BE(2)-C-shATF5-1 cells isolated 12 hours after intracardiac injection ±Dox (2 mg/mL) in drinking water. *ACTB* was used as an internal control.

To determine the molecular mechanism of ATF5-mediated anoikis resistance, we analyzed the expression of antiapoptotic BCL-2 family members BCL-2, BCL-XL, MCL-1, and proapoptotic family members BMF, BIM, BAD, BAX, and PUMA. ATF5 knockdown reduced BCL-2 and MCL-1 expression in adherent BE(2)-C and SK-N-DZ cells after 72 hours (Fig. 4A and B). In contrast, BCL-2 expression was reduced under suspension conditions, but no change in MCL-1 expression was observed (Fig. 4A and B). ATF5 knockdown increased BMF in suspension conditions but not in adherent cells. There was no difference in BCL-XL, BAD, BAX, BIM, or PUMA expression between suspension versus adherent conditions (Fig. 4A and B). These data demonstrate that ATF5 suppresses BMF in neuroblastoma only under suspension conditions. Consistent with the increase in protein levels, RT-PCR showed that ATF5 knockdown induced *BMF* mRNA in detached cells (Fig. 4C), suggesting that BMF upregulation was transcriptionally initiated. Next, we investigated whether ATF5 knockdown induces BMF in CTC *in vivo*. BE(2)-C-shATF5 cells with TF5 knocked down and isolated from mouse blood 12 hours after intracardiac injection exhibited induction of *BMF* mRNA (Fig. 4D). These findings suggest that ATF5 suppresses BMF transcription under nonadherent conditions *in vitro* and in CTC *in vivo*.

### BMF Regulates Neuroblastoma Anoikis

**FIGURE 5:**
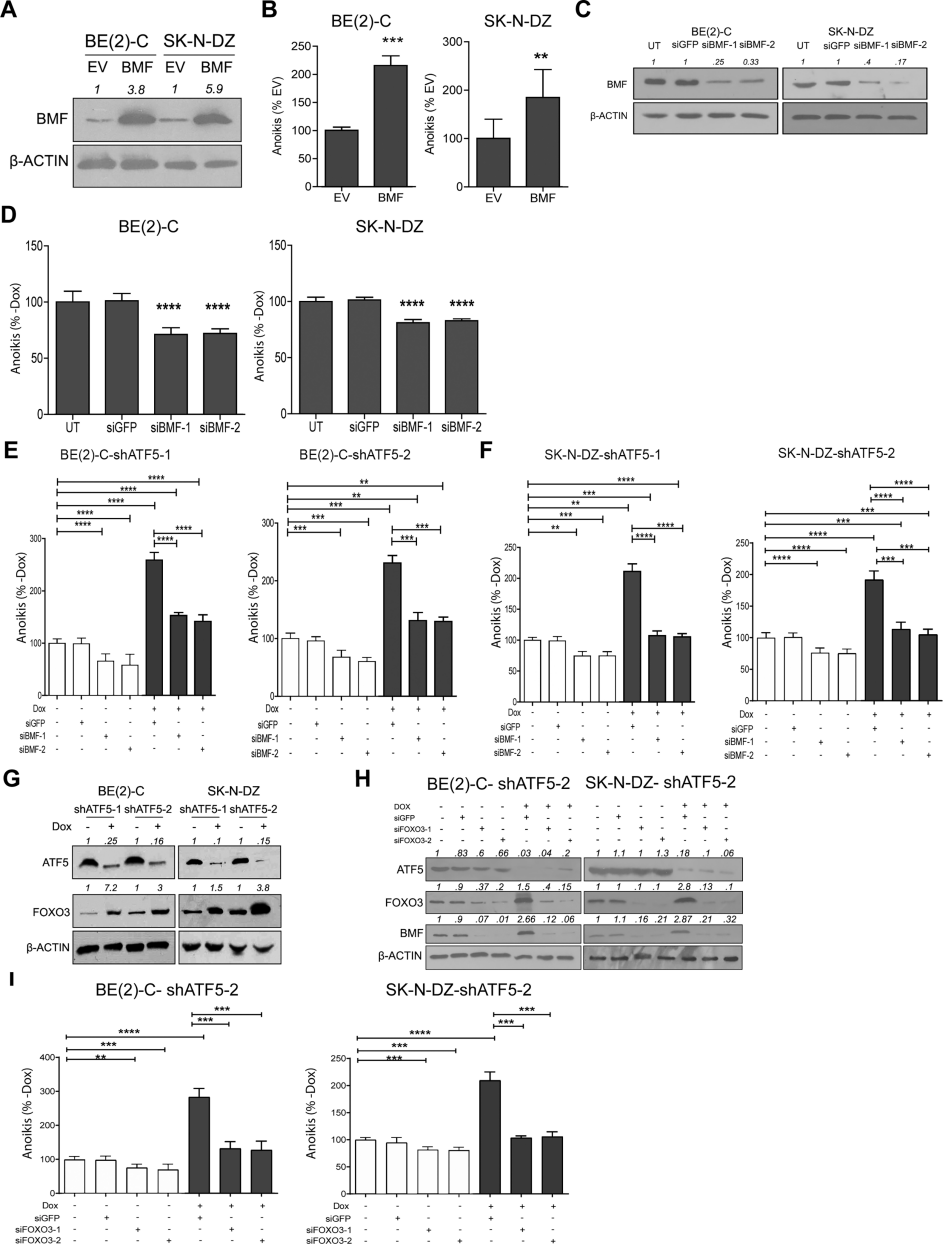
BMF is required for ATF5 depletion–dependent anoikis induction and is upregulated by FOXO3 induction after ATF5 knockdown. **A,** BMF expression, by immunoblot, in suspension cells 72 hours after transfection with empty vector (EV) or BMF expression vector in BE(2)-C cells and SK-N-DZ cells. β-Actin was used as a loading control. Densitometric analysis was performed using ImageJ. **B,** Quantification of anoikis 72 hours after transfection with BMF expression vector or EV in BE(2)-C and SK-N-DZ cells. **C,** Knockdown of BMF in BE(2)-C and SK-N-DZ cells. Adherent cells were transiently transfected with siGFP (negative control), siBMF-1, or siBMF-2. Twenty-four hours later, cells were seeded in nonadherent poly-HEMA–coated plates. A total of 96 hours after transfection, lysates were immunoblotted. Untreated (UT). β-Actin was used as a loading control. Densitometric analysis was performed using ImageJ. **D,** BMF knockdown suppresses anoikis in detached neuroblastoma cells. Quantification of anoikis in detached BE(2)-C and SK-N-DZ, 96 hours after transfection with siGFP (negative control), siBMF-1, or siBMF-2. BMF knockdown inhibits induction of anoikis following *ATF5* depletion**.** BE(2)-C (**E**) and SK-N-DZ (**F**), expressing shATF5-1 (left) or shATF5-2 (right), were transfected with siGFP (negative control), siBMF-1, or siBMF-2, and 24 hours later seeded in poly-HEMA–coated plates. Dox was added to deplete ATF5, and 72 hours later, anoikis was measured. −Dox (open bars), +Dox (closed bars). **G,** ATF5 knockdown in BE(2)-C and SK-N-DZ suspension cells elevates FOXO3 expression. Immunoblot analyses of ATF5 and FOXO3 in suspension cells expressing shATF5-1 or shATF5-2 at 72 hours ± Dox addition. β-Actin was used as a loading control. Densitometric analysis was performed using ImageJ. **H,** Immunoblot analyses show that FOXO3 knockdown with siRNA prevents BMF induction in response to ATF5 knockdown. BE(2)-C and SK-N-DZ cells, expressing shATF5-2, were transfected with siFOXO3-1 or siFOXO3-2 and 24 hours later were seeded in poly-HEMA–coated plates. Dox was added, and 72 hours later, cell lysates were immunoblotted. β-Actin was used as a loading control. Densitometric analysis was performed using ImageJ. **I,** Anoikis induction by ATF5 knockdown requires FOXO3. Percentage of BE(2)-C-shATF5-2 (left) and SK-N-DZ-shATF5-2 (right) cells undergoing anoikis at 72 hours, ± Dox addition and ± FOXO3 knockdown. −Dox (open bars), +Dox (closed bars). Cells were treated as described in **H**. Mean ± SD. **, *P* < 0.01; ***, *P* < 0.001; ****, *P* < 0.0001.

Because BMF has been previously linked to anoikis (8, 30), we investigated the role of BMF in neuroblastoma. BMF overexpression (Fig. 5A) markedly reduced the anchorage-independent viability of BE(2)-C and SK-N-DZ cells (Supplementary Fig. S8A) and significantly induced anoikis in BE(2)-C and SK-N-DZ cells (Fig. 5B). In contrast, BMF knockdown by two different siRNAs (Fig. 5C) significantly increased detached BE(2)-C and SK-N-DZ viability by an average of 30% and 25% (Supplementary Fig. S8B) and similarly reduced apoptosis (Fig. 5D). These data indicate that BMF regulates anoikis in neuroblastoma.

### BMF Mediates Induction of Anoikis Following ATF5 Depletion

We next addressed whether endogenous BMF mediates ATF5 depletion–induced anoikis. Adherent cells expressing inducible shATF5-1 or shATF5-2 were treated with either siBMF-1 or siBMF-2. After 24 hours, the cells were transferred to poly-HEMA plates (± Dox), and TUNEL assays were performed after 72 hours. ATF5 knockdown alone increased BE(2)-C apoptosis by an average of 125% compared with the controls, while BMF knockdown significantly reduced apoptosis (Fig. 5E). Moreover, BMF knockdown significantly inhibited anoikis induction in cells in which ATF5 was knocked down. Similar observations were made for SK-N-DZ cells in suspension, where anoikis was significantly lower in ATF5-depleted cells after BMF knockdown (Fig. 5F). Consistent with increased anoikis, BMF knockdown inhibited the ATF5 loss–induced reduction in BE(2)-C and SK-N-DZ anchorage-independent viability (Supplementary Fig. S9A and S9B). These results indicate that BMF is involved in the induction of anoikis caused by ATF5 knockdown.

### BMF Binds BCL-XL to Promote Anoikis

BMF has been reported to trigger anoikis by binding and inhibiting BCL-2 or BCL-XL (30, 31). We investigated whether BMF binds to BCL-XL after ATF5 knockdown. BCL-XL was not detected in the co-immunoprecipitated BMF samples of detached BE(2)-C-shATF5 and SK-N-DZ-shATF5 cells without Dox treatment (Supplementary Fig. S10). However, upon induction of BMF following ATF5 knockdown, BCL-XL was detected in the co-immunoprecipitated BMF samples. These findings suggest that, under suspension conditions, BMF does not detectably bind BCL-XL. However, following ATF5 depletion, BMF is upregulated and binds BCL-XL, triggering anoikis.

### ATF5 Downregulates BMF by Suppressing FOXO3 Expression

BMF has previously been shown to be a target of the transcription factor FOXO3 (32). ATF5 knockdown increased FOXO3 levels in suspended cells (Fig. 5G). FOXO3 knockdown, using two different siRNAs, abrogated the induction of BMF following ATF5 depletion (Fig. 5H). Furthermore, FOXO3 knockdown significantly reduced ATF5 loss–induced anoikis in BE(2)-C and SK-N-DZ cells (Fig. 5I). Consistent with these observations, FOXO3 knockdown rescued the ATF5 loss–induced reduction in BE(2)-C and SK-N-DZ anchorage-independent viability (Supplementary Fig. S11A and S11B). These data indicate that ATF5 suppresses BMF expression by downregulating FOXO3.

### CP-d/n-ATF5 Peptide Induces Anoikis Sensitivity

To target ATF5 therapeutically, we have developed CP-d/n-ATF5, a peptide that consists of a CP penetratin domain fused to a dominant-negative ATF5 sequence (CP-d/n-ATF5; ref. 16). *In vitro*, under adherent conditions, CP-d/n-ATF5 dose-dependently inhibited the growth of a panel of neuroblastoma cell lines, both *MYCN*-amplified and *MYCN*-non-amplified (Supplementary Fig. S12). Under suspension conditions, treatment with CP-d/n-ATF5 reduced anchorage-independent viability (Supplementary Fig. S13) and increased anoikis (Supplementary Fig. S14) in a dose-dependent manner, whereas penetratin (as control) treatment had no effect.

**FIGURE 6:**
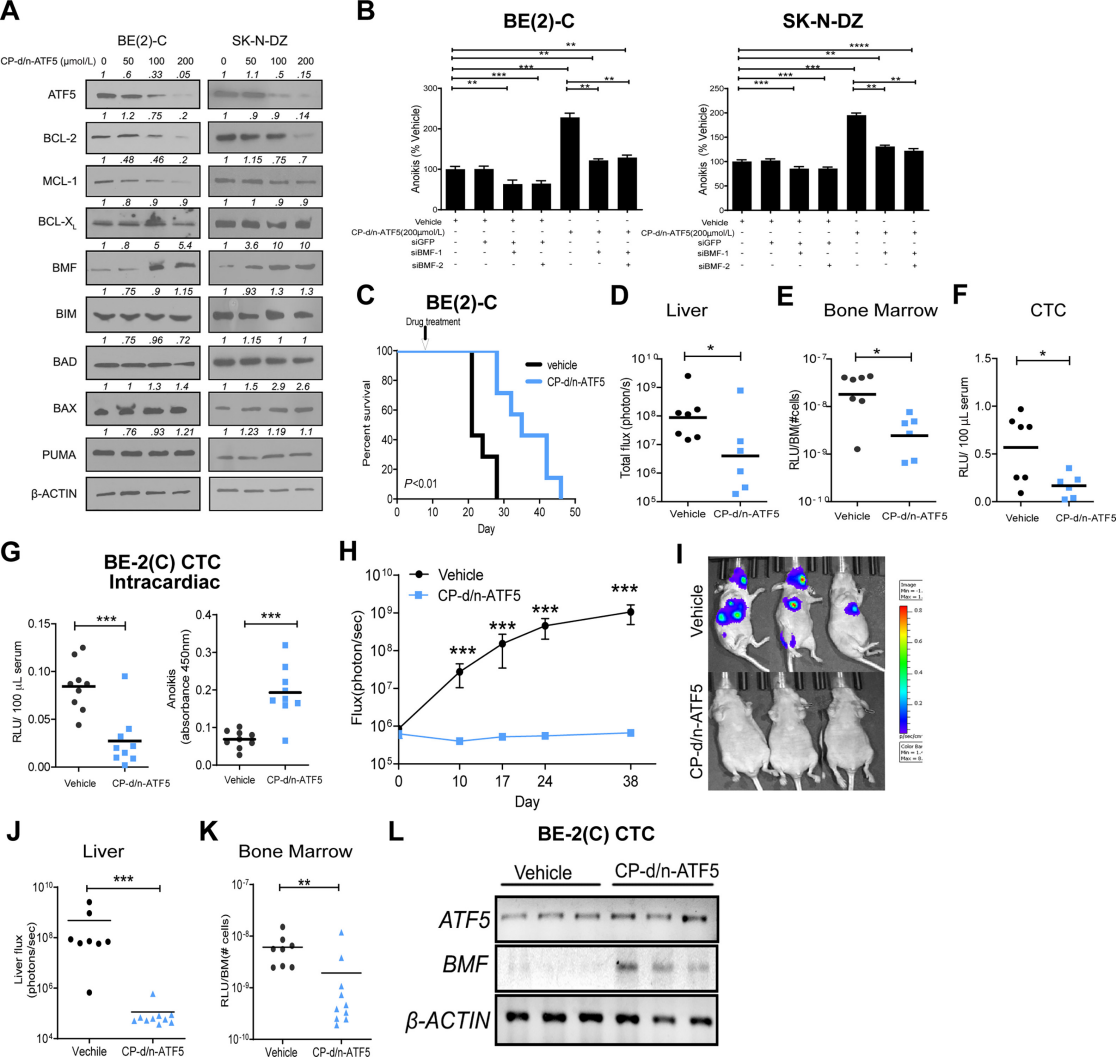
CP-d/n-ATF5 induces anoikis and inhibits neuroblastoma growth and metastasis *in vivo*. **A,** Effects of CP-dn-ATF5 on the expression of ATF5 and apoptosis-related BCL-2 proteins in suspension cells. Immunoblot of proapoptotic and antiapoptotic proteins in BE(2)-C and SK-N-DZ suspension cells at 72 hours after CP-d/n-ATF5 treatment. β-Actin was used as a loading control. Densitometric analysis was performed using ImageJ. **B,** BMF knockdown inhibits CP-d/n-ATF5–induced anoikis of BE(2)-C and SK-N-DZ cells. Adherent cells were treated with or without siBMFs (50 nmol/L) as indicated for 24 hours and then seeded in nonadherent plates. CP-d/n-ATF5 (200 µmol/L) was added, and 72 hours later, anoikis was evaluated. **C,** Kaplan–Meier analysis of survival of mice bearing BE(2)-C tumors, treated with vehicle (*n* = 7) or CP-d/n-ATF5 (50 mg/kg; *n* = 7). Treatment was started 7 days after cell implantation, once daily for the first 3 days, and then twice weekly. *P* < 0.01, log-rank (Mantel–Cox). **D,** Quantification of liver bioluminescence flux (photons/second) by *ex vivo* imaging at the time of euthanasia from mice bearing BE(2)-C tumors described in C. Vehicle (*n* = 7), CP-d/n-ATF5 (*n* = 6). **E,** Quantification of bioluminescence in bone marrow homogenate measured at the time of euthanasia (RLU/# BM cells, RLU: Relative luciferase unit). Vehicle (*n* = 7), CP-d/n-ATF5 (*n* = 6). **F,** Quantification of CTC measured at the time of euthanasia by bioluminescence of blood. Vehicle (*n* = 7), CP-d/n-ATF5 (*n* = 6). **G,** Twelve hours after intracardiac injection of BE(2)-C cells, blood was collected for (left) quantification of CTC by measurement of blood bioluminescence and (right) for measurement of apoptosis in isolated BE(2)-C CTC. Mice were treated with CP-d/n-ATF5 (*n* = 9) or vehicle (*n* = 9) immediately after intracardiac injection. **H,** BE(2)-C cells were injected intracardially and then treated with vehicle (*n* = 8) or CP-d/n-ATF5 (*n* = 10). Whole-body bioluminescence flux was then monitored for subsequent tumor cell growth. **I,** Representative bioluminescence images showing metastatic growth of BE(2)-C in mice treated with vehicle or CP-dn-ATF5 at day 38 of the experiment described in H. **J,** Quantification of total flux (photons/second) by *ex vivo* liver bioluminescence at time of euthanasia (day 38). **K,** Quantification of bioluminescence in bone marrow homogenate at the time of euthanasia (day 38). **L,** RT-PCR analysis of *ATF5*, *BMF,* and *ACTB* (internal control) in BE(2)-CTC from K. *, *P* < 0.05; **, *P* < 0.01; ***, *P* < 0.001.

CP-d/n-ATF5 depleted endogenous ATF5 in BE(2)-C and SK-N-DZ cells under suspension conditions (Fig. 6A) and under adherent conditions (Supplementary Fig. S15). CP-d/n-ATF5 treatment also reduced BCL-2 and MCL-1 expression under both conditions but induced BMF only in suspension culture (Fig. 6A). Moreover, CP-d/n-ATF5 treatment substantially increased anoikis compared with vehicle treatment in detached BE(2)-C and SK-N-DZ cells. In contrast, BMF knockdown inhibited this response (Fig. 6B). These findings indicate that BMF is an essential mediator of CP-d/n-ATF5–induced anoikis in neuroblastoma cells.

### CP-d/n-ATF5 Inhibits Tumor Growth and Metastases

We next evaluated whether CP-d/n-ATF5 could inhibit tumor growth and metastasis *in vivo*. Mice with established BE(2)-C and SK-N-DZ tumors were treated with CP-d/n-ATF5 or vehicle. Mice bearing BE(2)-C tumors treated with CP-d/n-ATF5 demonstrated decreased tumor growth as seen by bioluminescence at day 14 (Supplementary Fig. S16A). Survival was significantly increased, from a median of 22 days for vehicle to 38 days for animals receiving CP-d/n-ATF5 (Fig. 6C). CP-d/n-ATF5 treatment markedly decreased liver and bone marrow metastases with a 30-fold decrease in liver bioluminescence (Fig. 6D), and a 3-fold decrease in bioluminescence from bone marrow homogenates (Fig. 6E). CP-d/n-ATF5 treatment also resulted in a 2-fold decrease in CTC (Fig. 6F). Tumors examined at the time of euthanasia demonstrated increased apoptosis (Supplementary Fig. S16B). Similarly, CP-d/n-ATF5 significantly inhibited SK-N-DZ tumor growth over time (Supplementary Fig. S17A and S17B), with a >2-fold decrease in tumor weight at day 40 (Supplementary Fig. S17C). Moreover, CP-d/n-ATF5 reduced liver bioluminescence by 10-fold (Supplementary Fig. S17D), bone marrow bioluminescence by 3-fold (Supplementary Fig. S17E), and CTC by 7-fold (Supplementary Fig. S17F).

Finally, mice intracardially injected with BE(2)-C cells and treated with CP-d/n-ATF5 showed a marked reduction in viability and increased anoikis (∼3-fold) of CTC compared with vehicle-treated animals at 12 hours after treatment (Fig. 6G). In a separate cohort, CP-d/n-ATF5–treated mice exhibited minimal whole-body bioluminescence flux over time (Fig. 6H and I), minimal liver flux (Fig. 6J), and reduced bone marrow bioluminescence (Fig. 6K), indicating that metastatic growth was essentially abolished. Consistently, CP-d/n-ATF5 increased BMF mRNA levels in BE(2)-C CTC at 12 hours, although no change in ATF5 mRNA levels was observed (Fig. 6L). CP-d/n-ATF5 also caused a significant reduction in viability and increased anoikis of SK-N-DZ CTC at 12 hours (Supplementary Fig. S18). Collectively, these data indicate that CP-d/n-ATF5, by inducing BMF, renders CTCs vulnerable to anoikis and inhibits metastatic growth.

## Discussion

Using cell culture models and intrarenal spontaneous and intracardiac experimental metastasis models, we identified ATF5 as a critical regulator of neuroblastoma growth and metastasis. ATF5 modulation in multiple neuroblastoma cell lines altered anchorage-independent survival *in vitro* and *in vivo,* influencing their metastasizing ability. ATF5 has been linked to several adult cancers owing to its prosurvival function (10, 14, 17, 33). In our study, we strongly linked ATF5 expression to survival and metastasis.

The induction of SK-N-DZ and BE(2)-C anoikis was observed as early as 12 hours after ATF5 depletion *in vitro*. Similarly, anoikis of CTC was observed in mice 12 hours after ATF5 knockdown, indicating that ATF5 is a critical regulator of tumor cell survival in circulation. These findings also suggest that ATF5 has a short half-life in neuroblastoma cells, consistent with prior results that ATF5 has a 1-hour half-life in C6 glioma cells and a 45-minute half-life in HeLa cells (34).

ATF5 belongs to the bZIP family of transcription factors and has been implicated in the regulation of differentiation, protection from various cellular stresses, and tumor cell survival (10, 12, 14–17, 33). ATF5 knockdown promotes apoptosis in glioblastoma and breast cancer cells (17, 33, 35). Conversely, ATF5 overexpression protects tumor cells from apoptotic stimuli and promotes treatment resistance (34, 36, 37). Consistent with these findings, our study showed that knockdown ATF5 increased apoptotic cell death and inhibited neuroblastoma tumor growth, highlighting its antiapoptotic role in neuroblastoma. Prior studies showed that ATF5 suppresses apoptosis by downregulating intrinsic apoptotic pathway components BCL-2 and MCL-1 (27, 29). We found that ATF5 knockdown reduced the expression of BCL-2 and MCL-1, but not of BMF, in adherent neuroblastoma cells. However, in suspension conditions, depletion of BCL-2 and induction of BMF were observed, whereas MCL-1 remained unchanged. This suggests that ATF5-mediated anoikis resistance is mechanistically different from its antiapoptotic function in attached cells.

Anoikis is regulated by a delicate balance between proapoptotic and antiapoptotic protein levels (6). Increased proapoptotic proteins and inhibition of antiapoptotic proteins can shift the balance toward cell death. BMF, a BH3-only protein, regulates anoikis in epithelial cells (30). Under normal conditions, BMF is sequestered by binding to the myosin V-actin motor complex. Loss-of-cell attachment induces BMF release, which triggers apoptosis by binding and neutralizing prosurvival proteins (9, 30, 31). Our data showed that forced BMF expression in anoikis-resistant cells renders them vulnerable to anoikis. BMF knockdown prevented ATF5 depletion–induced anoikis. We also found that BMF binds to BCL-XL under suspension conditions. Furthermore, our data showed that ATF5 knockdown increased BMF in CTC, negatively affecting their circulation survival. We conclude that increased BMF is a rate-limiting factor driving neuroblastoma cell anoikis and inhibiting metastatic progression.

Our study therapeutically targeted ATF5 with a novel synthetic peptide inhibitor, CP-d/n-ATF5, which induces apoptosis in malignant cells but not normal cells (16, 37–39). We showed that CP-d/n-ATF5 induced anoikis and decreased anchorage-independent viability of several *MYCN*-amplified cell lines. CP-d/n-ATF5 also showed *in vivo* efficacy by inhibiting tumor growth, reducing CTC survival, and preventing metastatic progression. A single dose (50 mg/kg) of CP-d/n-ATF5 induced anoikis in circulating BE(2)-C and SK-N-DZ cells within 12 hours, indicating rapid CP-d/n-ATF5 activity. CP-d/n-ATF5 activity was specific, as the penetratin-only peptide showed no anoikis activity. Our findings suggest that BMF is a crucial mediator of CP-d/n-ATF5–induced anoikis. CP-d/n-ATF5 induced BMF in suspension culture but not in adherent cells. Loss of BMF prevented CP-d/n-ATF5–induced anoikis and decreased BMF was observed in the CTC of mice receiving CP-d/n-ATF5 treatments. CP-d/n-ATF5 treatment also caused the depletion of endogenous ATF5. Therefore, we speculate that CP-d/n-ATF5-induces the depletion of ATF5, leading to increased BMF, resulting in anoikis of neuroblastoma cells in suspension culture and the blood circulation of mice. Our studies identified CP-d/n-ATF5 as an effective therapeutic agent with antimetastatic efficacy and supported the clinical testing of such peptides in patients with neuroblastoma. Recent studies have identified the transcription factors CEBPB and CEBPD as CP-dn-ATF5 targets (40, 41), suggesting they are also potential targets for neuroblastoma.

A recent study has linked ATF5 with neuroblastoma cell survival (42). The study has shown that inhibition of protein arginine methyltransferases 1 (PRMT1) induces apoptosis of human neuroblastoma cells and that ATF5 acts as a downstream effector of PRMT1-mediated survival signaling. Overexpression of ATF5 rescues cell apoptosis triggered by PRMT1 inhibition. Here, we show that loss of ATF5 leads to neuroblastoma cell death. Our studies further focus on the mechanism downstream of ATF5, revealing ATF5 as a critical mediator of neuroblastoma metastasis, identifying BMF as a downstream effector of ATF5-mediated survival of CTC, and showing that drugging ATF5 with a CP peptide is effective in blocking neuroblastoma growth and metastasis.

As experimentally detailed in previous studies (43–45), ATF5 is highly expressed in neural stem cells and progenitors for neurons, astrocytes, and oligodendrocytes, in which it promotes proliferation and suppresses differentiation. In contrast, ATF5 downregulation or exposure to dn-ATF5 permits/accelerates their differentiation in the presence of appropriate trophic factors. In the case of neuroblastoma cells, we did not observe differentiation upon ATF5 depletion or CP-dn-ATF5 treatment, but rather the onset of apoptosis/anoikis. It remains to be seen whether such manipulations also promote neuronal differentiation of neuroblastoma cells under suitable conditions.

In conclusion, our study identified ATF5 as a promoter of neuroblastoma metastasis and CP-d/n-ATF5 as an antimetastatic therapeutic agent with the potential to improve the clinical outcomes of metastatic neuroblastoma.

## Supplementary Material

Supplementary Table 1 Supplementary Table 1. List of antibodies used in this study

Supplementary Figure 1 Supplementary Figure 1 shows that ATF5 is expressed in neuroblastoma cell lines.

Supplementary Figure 2 Supplementary Figure 2 shows that ATF5 knockdown decreases neuroblastoma cell viability and promotes apoptosis under adherent conditions

Supplementary Figure 3 Supplementary Figure 3 shows the decreased expression of ATF5 in + Dox tumors

Supplementary Figure 4 Supplementary Figure 4 shows that decreased ATF5 expression does not alter the invasiveness of BE(2)-C cells in vitro

Supplementary Figure 5 Supplementary Figure 5 shows that overexpression of ATF5 promotes anoikis resistance of CHLA-255 in vitro

Supplementary Figure 6 Supplementary Figure 6 shows a decrease in ATF5 after Dox addition under non-adherent suspension conditions.

Supplementary Figure 7 Supplementary Figure 7 shows that ATF5 knockdown decreases SK-N-DZ CTC survival

Supplementary Figure 8 Supplementary Figure S8 shows that BMF overexpression reduces the anchorage-independent viability of BE(2)-C and SK-N-DZ cells.

Supplementary Figure 9 Supplementary Figure 9 shows that BMF knockdown rescues ATF5 loss-induced reduction of anchorage-independent cell viability

Supplementary Figure 10 Supplementary Figure 10 shows that BMF binds to BCL-XL when ATF5 is depleted

Supplementary Figure 11 Supplementary Figure 11 shows that FOXO3 silencing rescues cell viability following ATF5 depletion

Supplementary Figure 12 Supplementary Figure 12 shows that CP-d/n-ATF5 inhibits the growth of MYCN-amplified and MYCN-non-amplified cell lines in a dose-dependent manner

Supplementary Figure 13 Supplementary Figure 13 shows that CP-d/n-ATF5 reduces the anchorage-independent viability of neuroblastoma cell lines

Supplementary Figure 14 Supplementary Figure 14 shows that CP-d/n-ATF5 induces anoikis of neuroblastoma cell lines

Supplementary Figure 15 Supplementary Figure 15 shows that CP-d/n-ATF5 treatment does not induce BMF under adherent conditions

Supplementary Figure 16 Supplementary Figure 16 shows that CP-d/n-ATF5 inhibits growth of BE(2)-C tumors and increases apoptosis

Supplementary Figure 17 Supplementary Figure 17 shows that CP-dn-ATF5 reduces tumor growth and metastasis of SK-N-DZ in vivo

Supplementary Figure 18 Supplementary Figure 18 shows that CP-d/n-ATF5 treatment decreased viability and induced anoikis of SK-N-DZ CTCs
