# Supplementary material for: Activating Transcription Factor 5 Promotes Neuroblastoma Metastasis by Inducing Anoikis Resistance
Source: Cancer Res Commun. 2023 Dec 12;3(12):2518–30. doi: 10.1158/2767-9764.CRC-23-0154 (PMC10714915; doi:10.1158/2767-9764.CRC-23-0154)
Supplement: Supplementary Table 1 — List of antibodies used in this study [file crc-23-0154-s01.docx]

| **Antibody** | **Source** | **Catalog#** |
| --- | --- | --- |
| ACTB | Cell Signaling Technology | 8457 |
| ATF5 | LSBio | LS-B5880-50 |
| BAD | Cell Signaling Technology | 9239 |
| BAK | Cell Signaling Technology | 6947 |
| BAX | Cell Signaling Technology | 5023 |
| BCL-2 | Cell Signaling Technology | 2872 |
| BCL-XL | Cell Signaling Technology | 2764 |
| BIM | Cell Signaling Technology | 2933 |
| BMF | Enzo | ALX-804-343-C100 |
| FOXO3 | Cell Signaling Technology | 2497 |
| MCL-1 | Cell Signaling Technology | 5453 |
| PUMA | Cell Signaling Technology | 12450 |

**Supplementary Table 1.** List of antibodies used in this study.
