# Supplementary material for: Activating Transcription Factor 5 Promotes Neuroblastoma Metastasis by Inducing Anoikis Resistance
Source: Cancer Res Commun. 2023 Dec 12;3(12):2518–30. doi: 10.1158/2767-9764.CRC-23-0154 (PMC10714915; doi:10.1158/2767-9764.CRC-23-0154)
Supplement: Supplementary Figure 1 — shows that ATF5 is expressed in neuroblastoma cell lines. [file crc-23-0154-s02.pdf]

Supplementary Figure 1

A

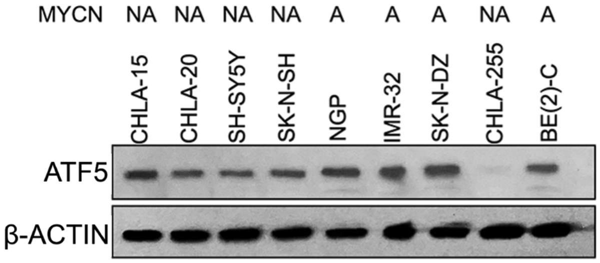

B

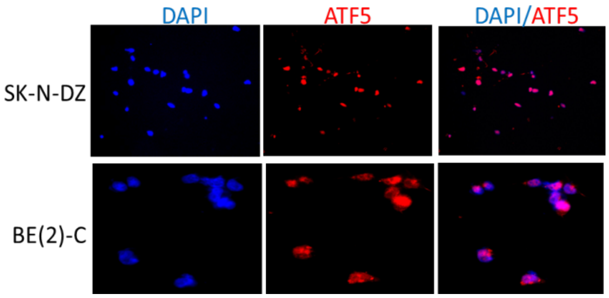

Supplementary Figure 1. ATF5 is expressed in neuroblastoma cell lines. (**A)** Western blot analysis for ATF5 in a panel of neuroblastoma cell lines, with *MYCN*-amplified (A) cell lines NGP, IMR-32, SK-N-DZ, BE(2)-C, and *MYCN*-non-amplified (NA) cell lines CHLA-15, CHLA-20, SH-SY5Y, SK-N-SH, CHLA-255. **(B)** Double immunofluorescence staining for ATF5 (red) and DAPI (blue) shows nuclear localization of ATF5 in SK-N-DZ and BE(2)-C cells.
