# Supplementary material for: Activating Transcription Factor 5 Promotes Neuroblastoma Metastasis by Inducing Anoikis Resistance
Source: Cancer Res Commun. 2023 Dec 12;3(12):2518–30. doi: 10.1158/2767-9764.CRC-23-0154 (PMC10714915; doi:10.1158/2767-9764.CRC-23-0154)
Supplement: Supplementary Figure 2 — shows that ATF5 knockdown decreases neuroblastoma cell viability and promotes apoptosis under adherent conditions [file crc-23-0154-s03.pdf]

Supplementary Figure 2

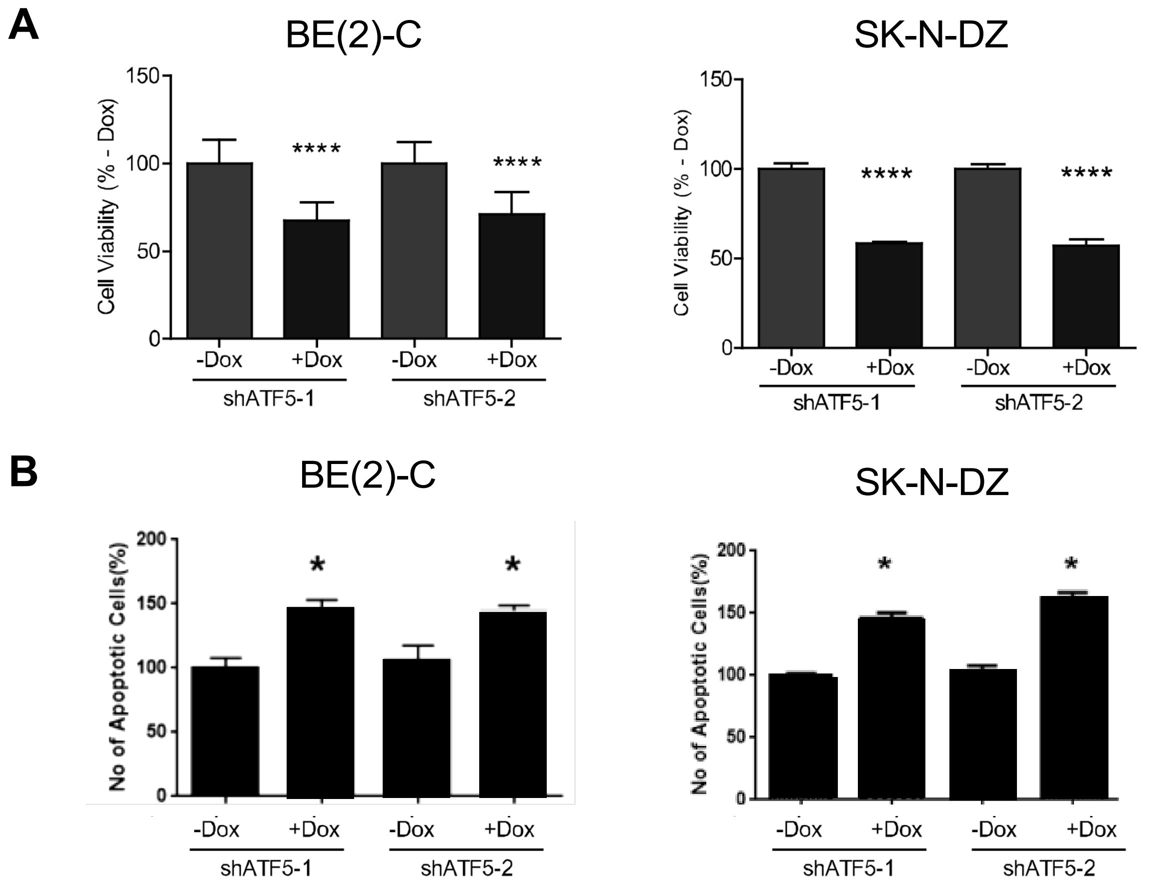

Supplementary Figure 2. ATF5 knockdown decreases neuroblastoma cell viability and promotes apoptosis under adherent conditions. Neuroblastoma cell lines were infected with two different Dox-inducible shRNAs against ATF5. **(A)** Quantification of cell viability, measured by CCK-8 assay, of BE(2)-C (left) and SK-N-DZ (right) cells 72 hours after 1 μg/ml doxycycline treatment under adherent conditions. Experiments were performed in triplicate. **(B)** Quantification of apoptotic cell death measured by TUNEL staining at 72 hours after Dox addition. Mean ± std dev. *, $P < 0.05$; ***, $P < 0.001$.
