# Supplementary material for: Activating Transcription Factor 5 Promotes Neuroblastoma Metastasis by Inducing Anoikis Resistance
Source: Cancer Res Commun. 2023 Dec 12;3(12):2518–30. doi: 10.1158/2767-9764.CRC-23-0154 (PMC10714915; doi:10.1158/2767-9764.CRC-23-0154)
Supplement: Supplementary Figure 3 — shows the decreased expression of ATF5 in + Dox tumors [file crc-23-0154-s04.pdf]

Supplementary Figure 3

A

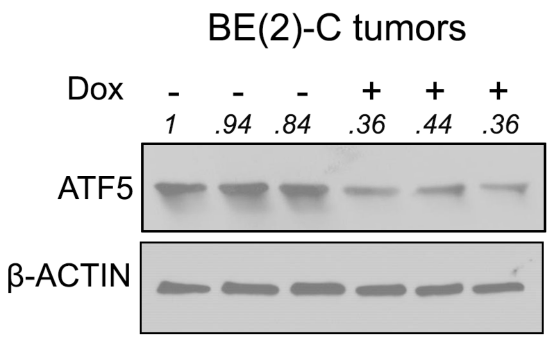

B

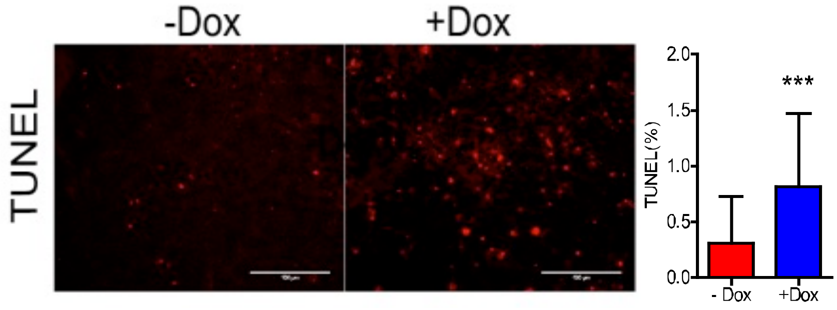

Supplementary Figure 3. Decreased expression of ATF5 in + Dox tumors. (A) Immunoblot analysis of ATF5 in -Dox and +Dox BE(2)-C-shATF5-2 tumors, demonstrating decreased ATF5 in +Dox tumors. Densitometric analysis was performed using ImageJ. **(B)** Representative images and quantification of TUNEL staining as % area of -Dox and +Dox tumors. Mean ± std dev ***, $P < 0.001$.
