# Supplementary material for: Activating Transcription Factor 5 Promotes Neuroblastoma Metastasis by Inducing Anoikis Resistance
Source: Cancer Res Commun. 2023 Dec 12;3(12):2518–30. doi: 10.1158/2767-9764.CRC-23-0154 (PMC10714915; doi:10.1158/2767-9764.CRC-23-0154)
Supplement: Supplementary Figure 4 — shows that decreased ATF5 expression does not alter the invasiveness of BE(2)-C cells in vitro [file crc-23-0154-s05.pdf]

Supplementary Figure 4

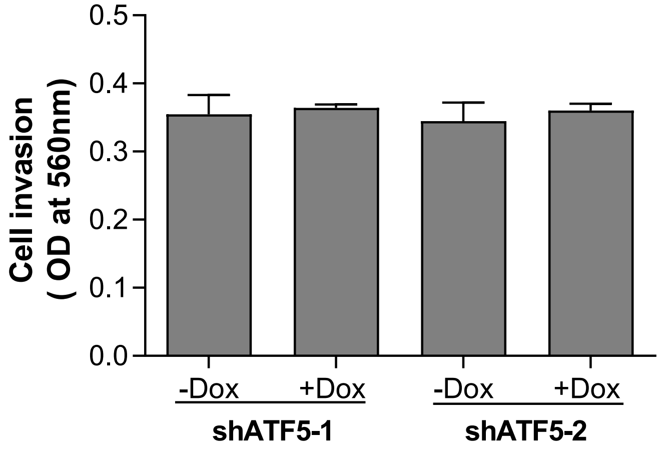

Supplementary Figure 4. Decreased ATF5 expression does not alter the invasiveness of BE(2)-C cells *in vitro*. BE(2)-C-shATF5-1 cells and BE(2)-C-shATF5-2 cells ($4x10^5$ cells/well) were seeded in serum-free medium in the upper chamber of CytoSelect™ 24-Well Cell Invasion plate (8µm pore-size, CellBiolabs) inserts. Complete medium was added to the lower chamber. Dox (1 µg/ml) was added as indicated, and 72 hours later, non-invasive cells were removed by swabbing the interior of the inserts with a cotton-tipped swab. Inserts were washed, incubated with extraction solution for 10 minutes, and absorbance was measured at 560 nm in a plate reader.
