# Supplementary material for: Activating Transcription Factor 5 Promotes Neuroblastoma Metastasis by Inducing Anoikis Resistance
Source: Cancer Res Commun. 2023 Dec 12;3(12):2518–30. doi: 10.1158/2767-9764.CRC-23-0154 (PMC10714915; doi:10.1158/2767-9764.CRC-23-0154)
Supplement: Supplementary Figure 5 — shows that overexpression of ATF5 promotes anoikis resistance of CHLA-255 in vitro [file crc-23-0154-s06.pdf]

Supplementary Figure 5

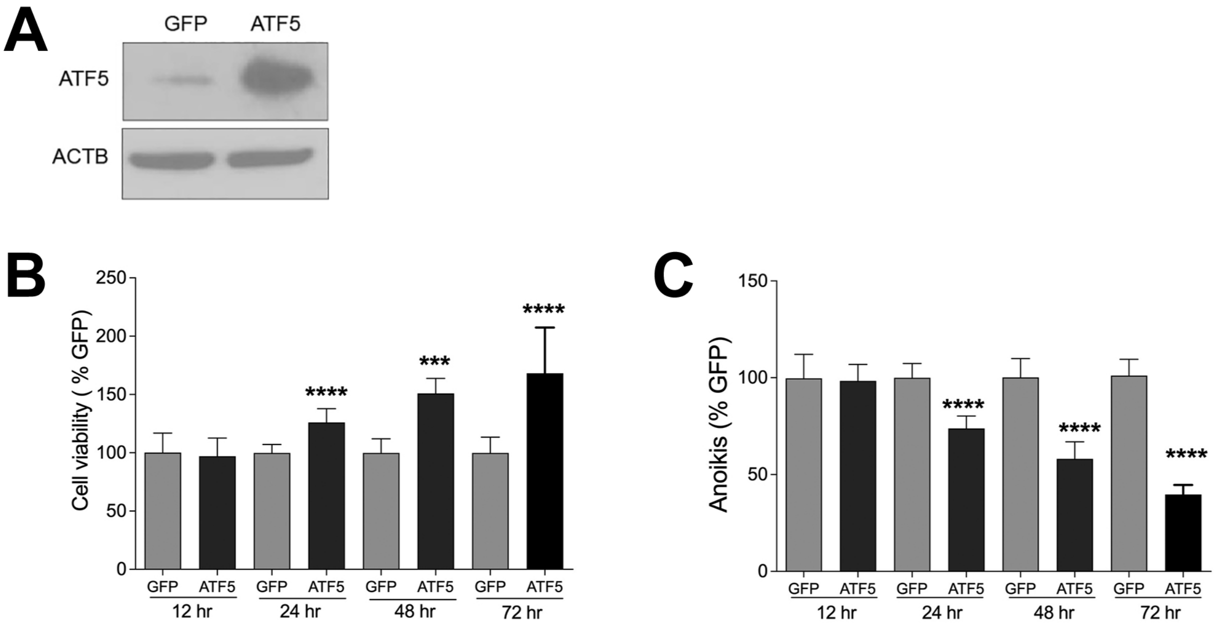

Supplementary Figure 5. Overexpression of ATF5 promotes anoikis resistance of CHLA-255 *in vitro*. (A) ATF5 overexpression, by immunoblot, in suspension culture of CHLA-255 cells, 72 hours after transient transfection with pCCL-GFP or pCCL-ATF5. β-Actin was used as a loading control. CHLA-255 suspension **(B)** cell viability and **(C)** anoikis at 12, 24, 48, and 72 hours after transfection. At each time point, the comparison is between cells overexpressing ATF5 and control cells expressing GFP (100%). Mean ± SD. **, $P<0.01$; ***, $P<0.001$; ****, $P<0.0001$
