# Supplementary material for: Activating Transcription Factor 5 Promotes Neuroblastoma Metastasis by Inducing Anoikis Resistance
Source: Cancer Res Commun. 2023 Dec 12;3(12):2518–30. doi: 10.1158/2767-9764.CRC-23-0154 (PMC10714915; doi:10.1158/2767-9764.CRC-23-0154)
Supplement: Supplementary Figure 6 — shows a decrease in ATF5 after Dox addition under non-adherent suspension conditions. [file crc-23-0154-s07.pdf]

Supplementary Figure 6

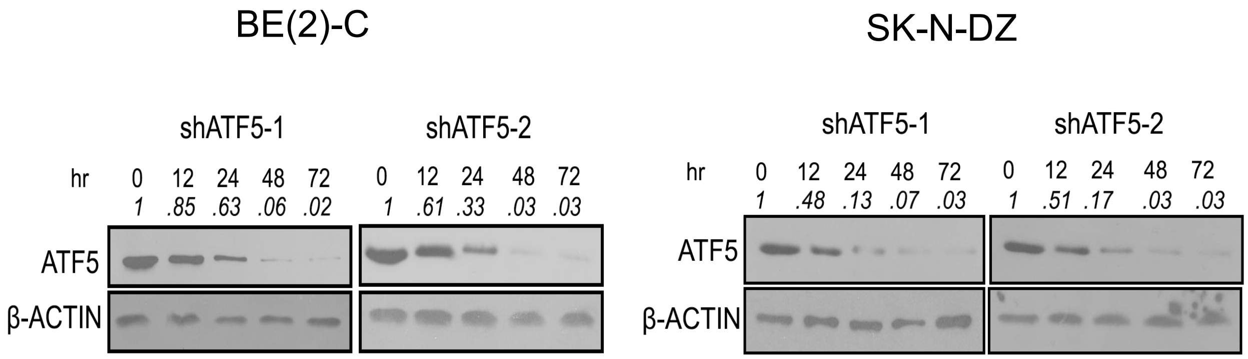

Supplementary Figure 6. Decrease in ATF5 after Dox addition under non-adherent suspension culture. Immunoblot analyses of ATF5 knockdown in BE(2)-C and SK-N-DZ cells expressing Dox-inducible shATF5-1 and shATF5-2 at various times after Dox addition. Densitometric analysis was performed using ImageJ.
