# Supplementary material for: Activating Transcription Factor 5 Promotes Neuroblastoma Metastasis by Inducing Anoikis Resistance
Source: Cancer Res Commun. 2023 Dec 12;3(12):2518–30. doi: 10.1158/2767-9764.CRC-23-0154 (PMC10714915; doi:10.1158/2767-9764.CRC-23-0154)
Supplement: Supplementary Figure 7 — shows that ATF5 knockdown decreases SK-N-DZ CTC survival [file crc-23-0154-s08.pdf]

Supplementary Figure 7

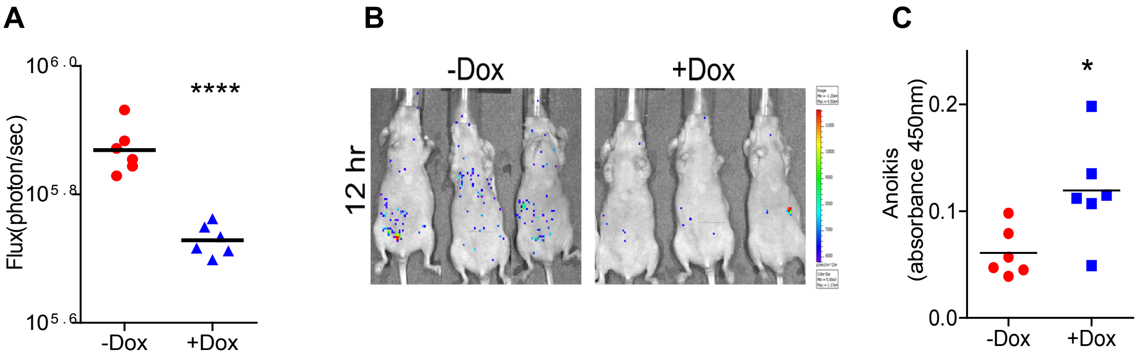

Supplementary Figure 7. ATF5 knockdown decreases SK-N-DZ CTC survival. (A) Quantification of mouse whole body bioluminescence flux (photon/sec) 12 hours after intracardiac injection of SK-N-DZ-shATF5-2 cells. Mice received +Dox (n=6) in drinking water for 3 days before injection or -Dox (n=6). **(B)** Representative bioluminescence images from mice at 12 hours after SK-N-DZ-shATF5-2 cell injection -Dox or +Dox. **(C)** Quantification of apoptosis of SK-N-DZ-shATF5-2 CTC isolated from mice 12 hours after intracardiac injection -Dox or +Dox. *P<0.05, ****, P < 0.0001.
