# Supplementary material for: Activating Transcription Factor 5 Promotes Neuroblastoma Metastasis by Inducing Anoikis Resistance
Source: Cancer Res Commun. 2023 Dec 12;3(12):2518–30. doi: 10.1158/2767-9764.CRC-23-0154 (PMC10714915; doi:10.1158/2767-9764.CRC-23-0154)
Supplement: Supplementary Figure 8 — Supplementary Figure S8 shows that BMF overexpression reduces the anchorage-independent viability of BE(2)-C and SK-N-DZ cells. [file crc-23-0154-s09.pdf]

Supplementary Figure 8

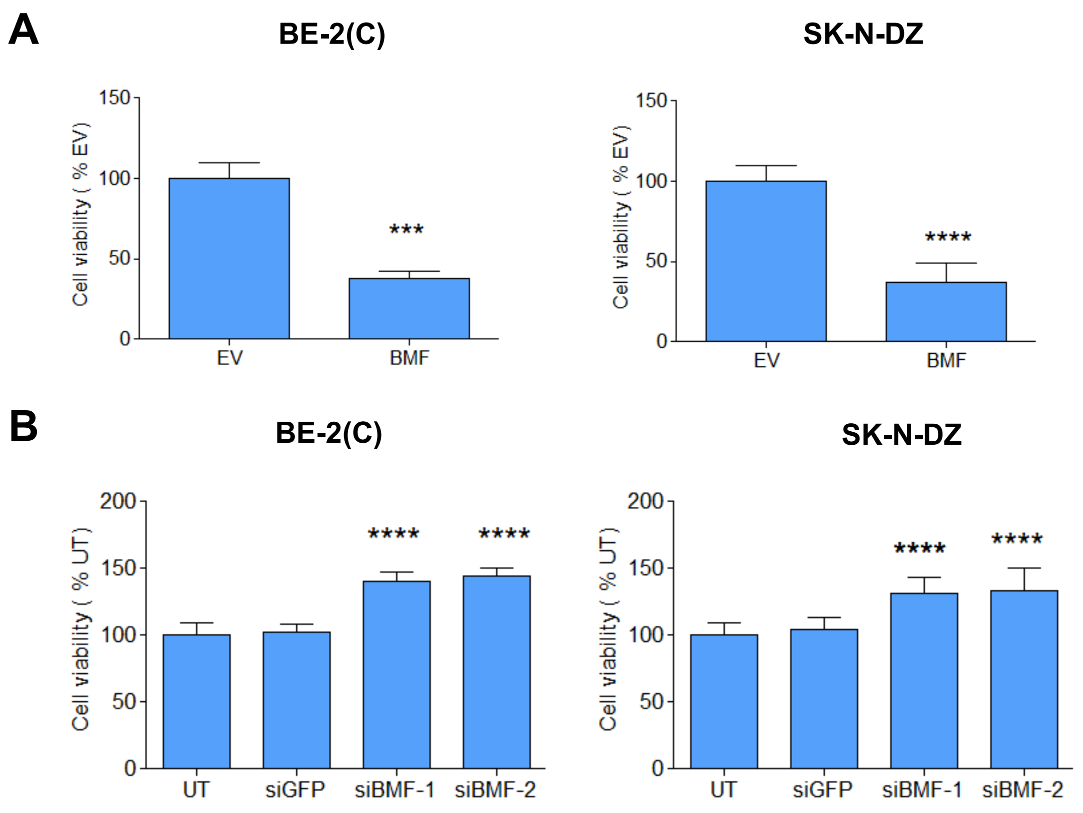

Supplementary Figure 8. BMF overexpression reduces the anchorage-independent viability of BE(2)-C and SK-N-DZ cells. (A) Quantification of cell viability of BE(2)-C and SK-N-DZ cells in suspension culture 72 hours after transient transfection with EV (empty) and BMF expression vectors. Mean ± std dev. **(B)** Quantification of cell viability of detached BE(2)-C and SH-SY5Y cells 96 hours after siGFP and siBMF treatment. Mean ± std dev. ***, $P < 0.001$; ****, $P < 0.0001$. Attached cells were treated with siRNAs for 24 hours and then seeded in a poly-HEMA coated plate. Viabilities of suspension cells were measured 72 hours later.
