# Supplementary material for: Activating Transcription Factor 5 Promotes Neuroblastoma Metastasis by Inducing Anoikis Resistance
Source: Cancer Res Commun. 2023 Dec 12;3(12):2518–30. doi: 10.1158/2767-9764.CRC-23-0154 (PMC10714915; doi:10.1158/2767-9764.CRC-23-0154)
Supplement: Supplementary Figure 9 — shows that BMF knockdown rescues ATF5 loss-induced reduction of anchorage-independent cell viability [file crc-23-0154-s10.pdf]

Supplementary Figure 9

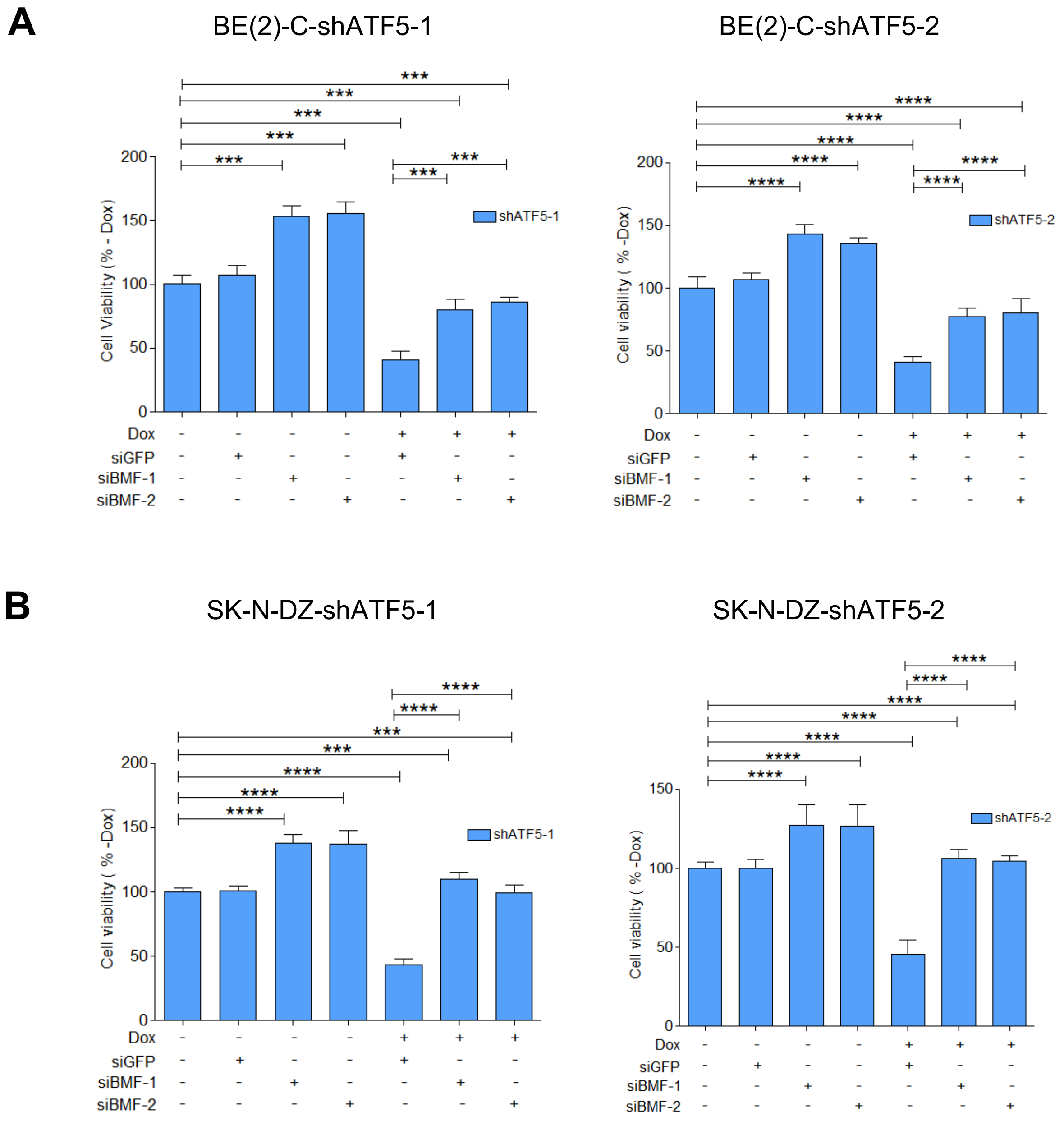

Supplementary Figure 9. BMF knockdown rescues ATF5 loss-induced reduction of anchorage-independent cell viability. (A) Adherent BE(2)-C cells, expressing shATF5-1 (left) or shATF5-2 (right), were treated with siBMFs for 24 hours, and then the cells were seeded in poly-HMEA coated plates. Dox was added to induce ATF5 knockdown. 72 hours after Dox addition, cell viability was measured by CCK-8 assay. **(B)** Quantification of cell viability in SK-N-

DZ cells following siBMF and Dox treatments. Cells were treated as described in A. Mean ± std dev. ***, $P<0.001$; ****, $P<0.0001$.
