# Supplementary material for: Activating Transcription Factor 5 Promotes Neuroblastoma Metastasis by Inducing Anoikis Resistance
Source: Cancer Res Commun. 2023 Dec 12;3(12):2518–30. doi: 10.1158/2767-9764.CRC-23-0154 (PMC10714915; doi:10.1158/2767-9764.CRC-23-0154)
Supplement: Supplementary Figure 10 — shows that BMF binds to BCL-XL when ATF5 is depleted [file crc-23-0154-s11.pdf]

Supplementary Figure 10

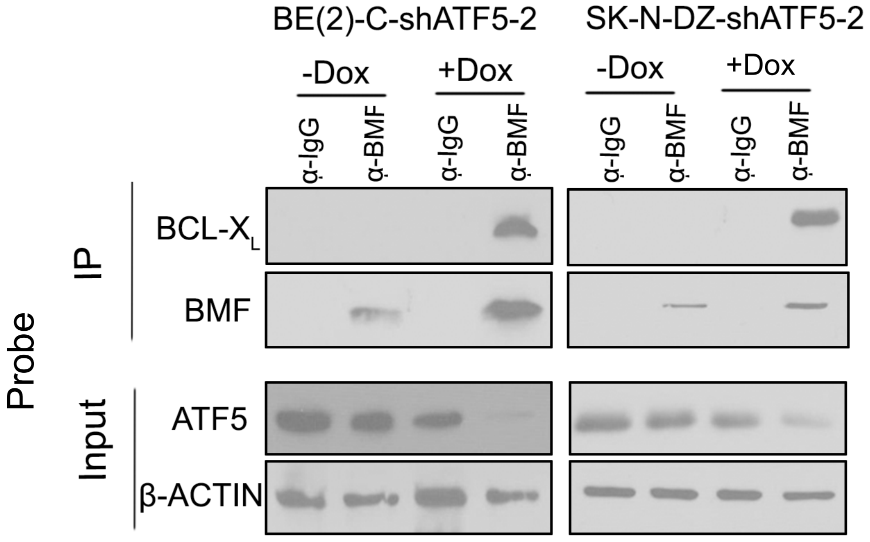

Supplementary Figure 10. BMF binds to BCL-XL when ATF5 is depleted. Top Panel: BE(2)-C and SK-N-DZ suspension cells, expressing shATF5-2, were treated with or without Dox for 72 hours. Cell lysates were subjected to co-immunoprecipitation with α-BMF antibody or nonspecific IgG. Immunoprecipitated samples were analyzed by immunoblot for BCL-XL and BMF. BCL-XL was detected in immunoprecipitated samples with α-BMF antibody after Dox treatment. Bottom Panel: Inputs for co-immunoprecipitation were also subjected to immunoblot analysis for ATF5 and β-ACTIN to confirm ATF5 knockdown.
