# Supplementary material for: Activating Transcription Factor 5 Promotes Neuroblastoma Metastasis by Inducing Anoikis Resistance
Source: Cancer Res Commun. 2023 Dec 12;3(12):2518–30. doi: 10.1158/2767-9764.CRC-23-0154 (PMC10714915; doi:10.1158/2767-9764.CRC-23-0154)
Supplement: Supplementary Figure 11 — shows that FOXO3 silencing rescues cell viability following ATF5 depletion [file crc-23-0154-s12.pdf]

Supplementary Figure 11

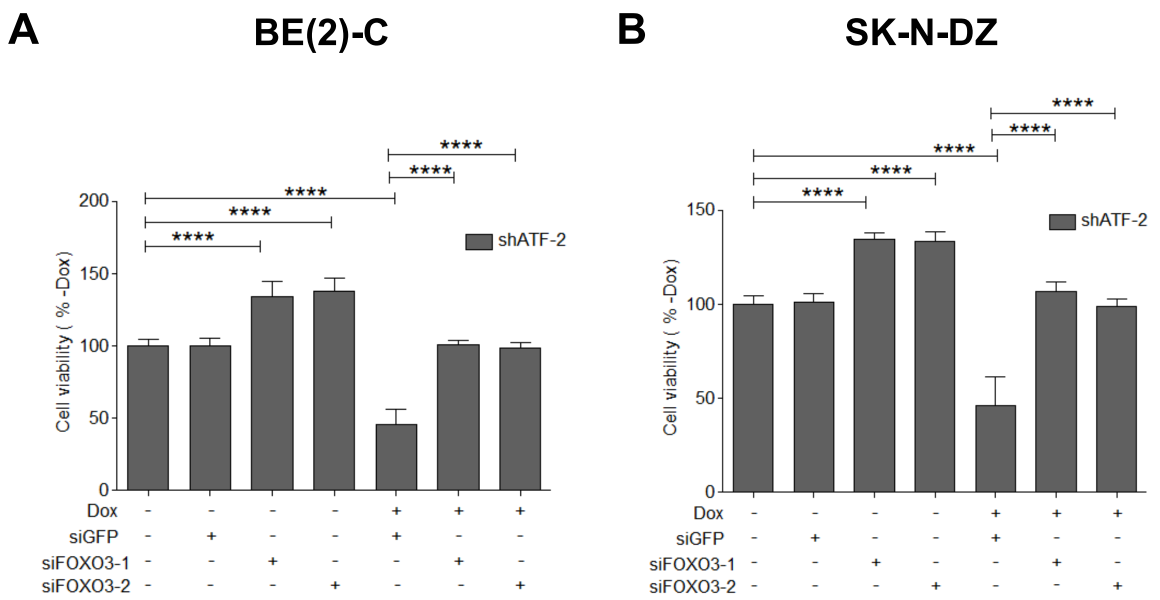

Supplementary Figure 11. FOXO3 silencing rescues cell viability following ATF5 depletion. (A) BE(2)-C-shATF5-2 cells and **(B)** SK-N-DZ-sh-ATF5-2 cells were transfected with siFOXO3-1 or siFOXO3-2 and, 24 hours later, were seeded in poly-HEMA coated plates. Dox was added or not, and 72 hours later, cell viability was measured by CCK-8 assay. Mean ± std dev. ****, $P<0.0001$.
