# Supplementary material for: Activating Transcription Factor 5 Promotes Neuroblastoma Metastasis by Inducing Anoikis Resistance
Source: Cancer Res Commun. 2023 Dec 12;3(12):2518–30. doi: 10.1158/2767-9764.CRC-23-0154 (PMC10714915; doi:10.1158/2767-9764.CRC-23-0154)
Supplement: Supplementary Figure 12 — shows that CP-d/n-ATF5 inhibits the growth of MYCN-amplified and MYCN-non-amplified cell lines in a dose-dependent manner [file crc-23-0154-s13.pdf]

Supplementary Figure 12

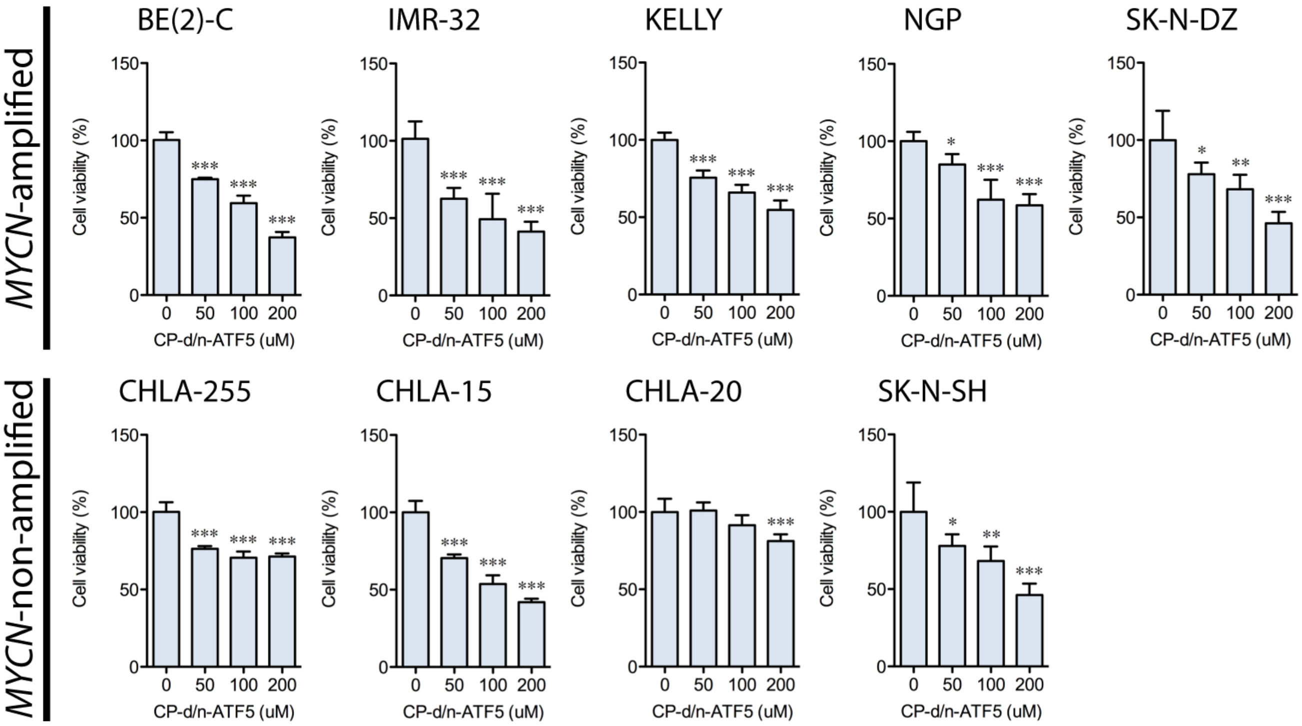

Supplementary Figure 12. CP-d/n-ATF5 inhibits the growth of *MYCN*-amplified and *MYCN*-non-amplified cell lines in a dose-dependent manner. Adherent cells were treated with vehicle, or 50, 100, or 200 μM CP-d/n-ATF5 for 72 hours, and cell viability was measured by CCK8 assay. *, $P<0.05$; **, $P<0.01$; ***, $P<0.001$.
