# Supplementary material for: Activating Transcription Factor 5 Promotes Neuroblastoma Metastasis by Inducing Anoikis Resistance
Source: Cancer Res Commun. 2023 Dec 12;3(12):2518–30. doi: 10.1158/2767-9764.CRC-23-0154 (PMC10714915; doi:10.1158/2767-9764.CRC-23-0154)
Supplement: Supplementary Figure 14 — shows that CP-d/n-ATF5 induces anoikis of neuroblastoma cell lines [file crc-23-0154-s15.pdf]

Supplementary Figure 14

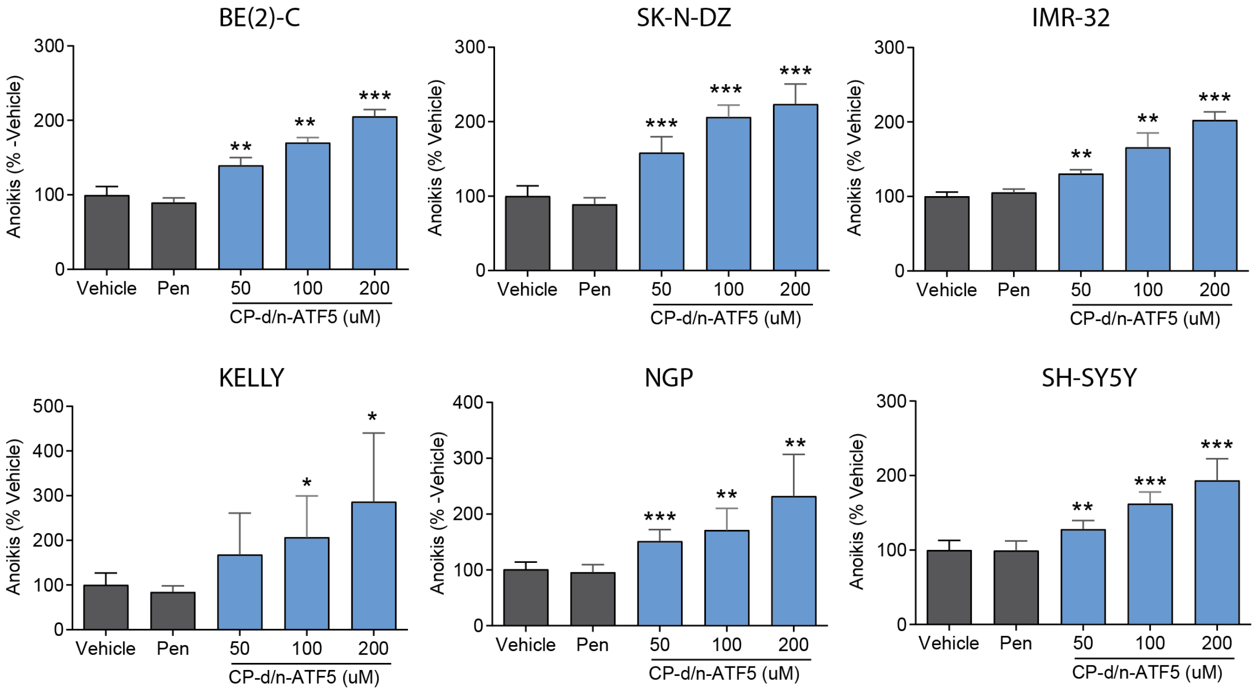

Supplementary Figure 14. CP-d/n-ATF5 induces anoikis of neuroblastoma cell lines. BE(2)-C, SK-N-DZ, IMR-32, KELLY, NGP and SH-SY5Y cells under suspension conditions were treated with vehicle, penetratin (Pen, 200 μM) or 50, 100, or 200 μM CP-d/n-ATF5 for 72 hours, and anoikis was measured by TUNEL assay. Mean ± std dev. *, $P<0.05$; **, $P<0.01$; ***, $P<0.001$.
