# Supplementary material for: Activating Transcription Factor 5 Promotes Neuroblastoma Metastasis by Inducing Anoikis Resistance
Source: Cancer Res Commun. 2023 Dec 12;3(12):2518–30. doi: 10.1158/2767-9764.CRC-23-0154 (PMC10714915; doi:10.1158/2767-9764.CRC-23-0154)
Supplement: Supplementary Figure 15 — shows that CP-d/n-ATF5 treatment does not induce BMF under adherent conditions [file crc-23-0154-s16.pdf]

Supplementary Figure 15

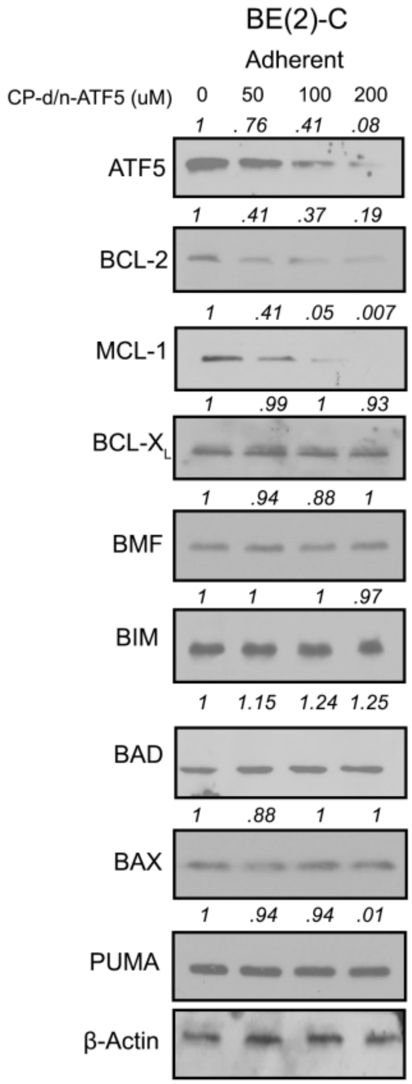

Supplementary Figure 15. CP-d/n-ATF5 treatment does not induce BMF under adherent conditions. BE(2)-C cells under adherent conditions were treated with vehicle, or 50, 100, or 200 μM CP-d/n-ATF5 for 72 hours. Immunoblot of pro-apoptotic and anti-apoptotic proteins was then performed. Densitometric analysis was conducted using ImageJ.
