# Supplementary material for: Activating Transcription Factor 5 Promotes Neuroblastoma Metastasis by Inducing Anoikis Resistance
Source: Cancer Res Commun. 2023 Dec 12;3(12):2518–30. doi: 10.1158/2767-9764.CRC-23-0154 (PMC10714915; doi:10.1158/2767-9764.CRC-23-0154)
Supplement: Supplementary Figure 16 — shows that CP-d/n-ATF5 inhibits growth of BE(2)-C tumors and increases apoptosis [file crc-23-0154-s17.pdf]

Supplementary Figure 16

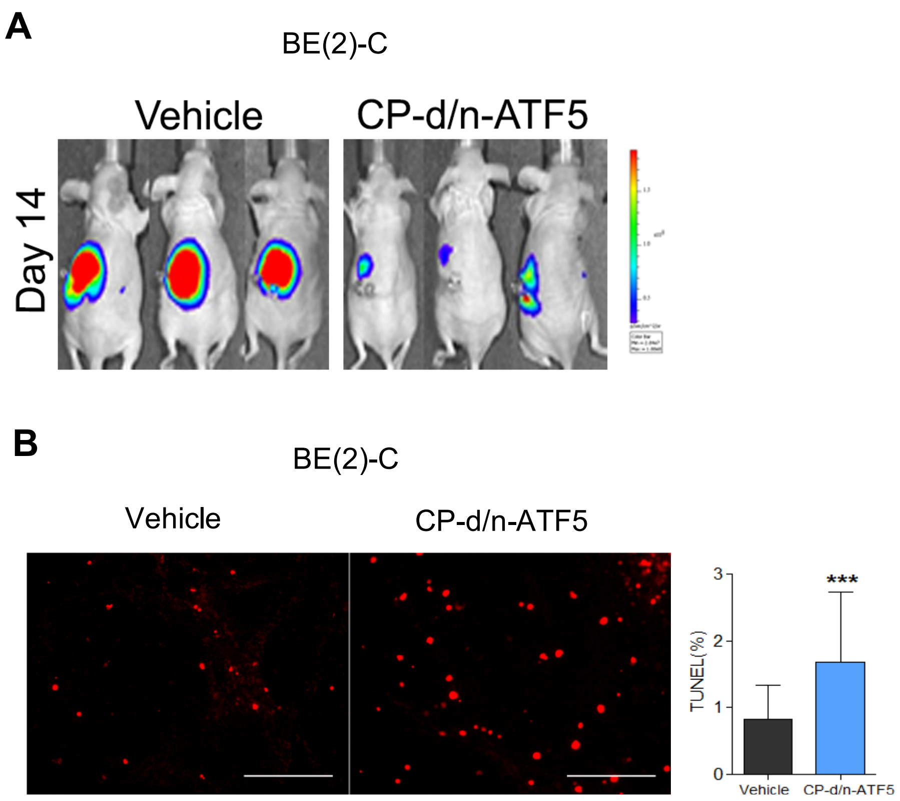

Supplementary Figure 16. CP-d/n-ATF5 inhibits growth of BE(2)-C tumors and increases apoptosis. (A) Representative bioluminescence at day 14 of mice with BE(2)-C tumors treated with vehicle or CP-d/n-ATF5 (50 mg/kg). **(B)** Immunofluorescence images of TUNEL staining of tumors treated with vehicle or CP-d/n-ATF5. TUNEL-positive cells were quantified as described. ***, $P<0.001$
