# Supplementary material for: Activating Transcription Factor 5 Promotes Neuroblastoma Metastasis by Inducing Anoikis Resistance
Source: Cancer Res Commun. 2023 Dec 12;3(12):2518–30. doi: 10.1158/2767-9764.CRC-23-0154 (PMC10714915; doi:10.1158/2767-9764.CRC-23-0154)
Supplement: Supplementary Figure 17 — shows that CP-dn-ATF5 reduces tumor growth and metastasis of SK-N-DZ in vivo [file crc-23-0154-s18.pdf]

Supplementary Figure 17

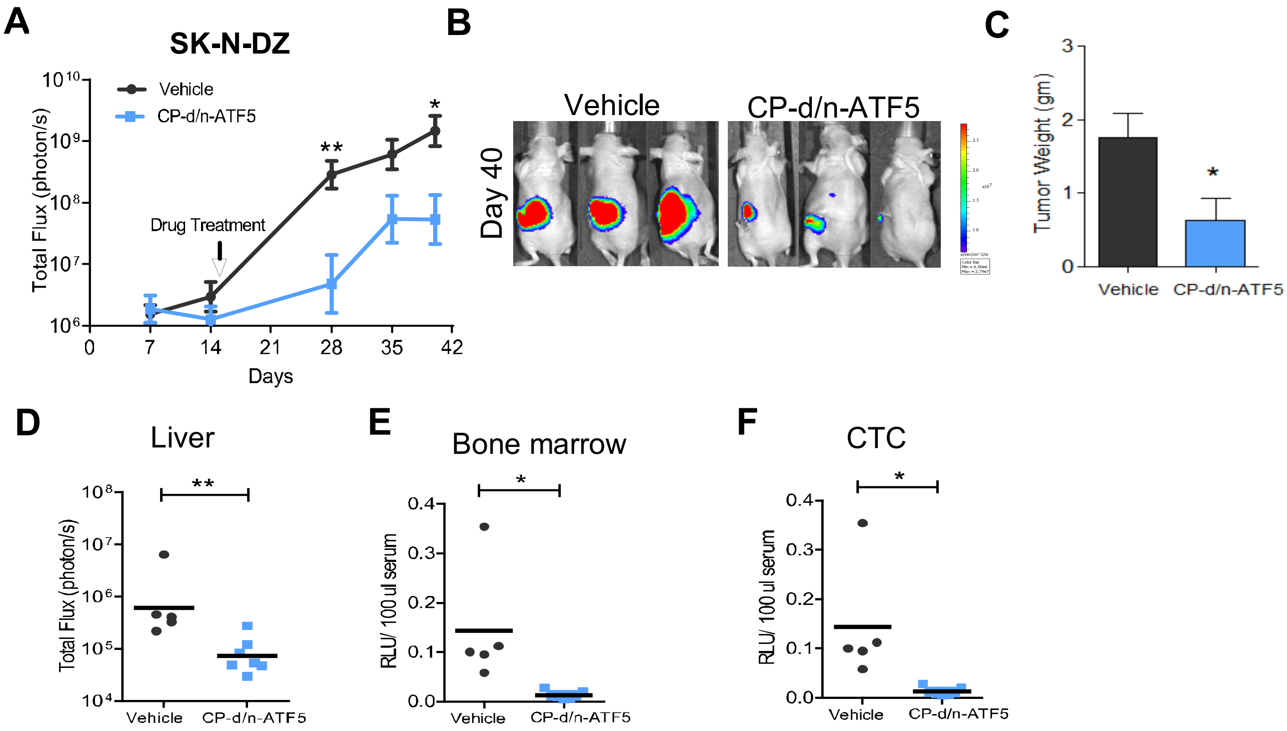

Supplementary Figure 17. CP-dn-ATF5 reduces tumor growth and metastasis of SK-N-DZ *in vivo.* **(A)** Bioluminescence flux (photons/sec), indicative of tumor growth of SK-N-DZ tumors at various times after initiation and treatment with vehicle (n=5) or CP-d/n-ATF5 (n=7). Treatment with CP-d/n-ATF5 (50 mg/kg) or vehicle was by IP injection, starting at day 14, once per day for the first three days and then twice per week. **(B)** Representative bioluminescence images of mice with SK-N-DZ tumors treated with vehicle or CP-d/n-ATF5 at day 40. (**C**) All mice were euthanatized at day 40, with Quantification of the weight of SK-N-DZ tumors, measured at the time of euthanasia, treated with vehicle or CP-d/n-ATF5. (**D**) Quantification of total flux (photon/sec) by *ex vivo* liver bioluminescence from mice at the time of euthanasia in the experiment described in panel (A). (**E**) Quantification of bioluminescence in bone marrow homogenate from mice at the time of euthanasia. (**F**) Quantification of bioluminescence in blood from mice at the time of euthanasia. *, $P<0.05$; **, $P<0.01$
