# Supplementary material for: Activating Transcription Factor 5 Promotes Neuroblastoma Metastasis by Inducing Anoikis Resistance
Source: Cancer Res Commun. 2023 Dec 12;3(12):2518–30. doi: 10.1158/2767-9764.CRC-23-0154 (PMC10714915; doi:10.1158/2767-9764.CRC-23-0154)
Supplement: Supplementary Figure 18 — shows that CP-d/n-ATF5 treatment decreased viability and induced anoikis of SK-N-DZ CTCs [file crc-23-0154-s19.pdf]

Supplementary Figure 18

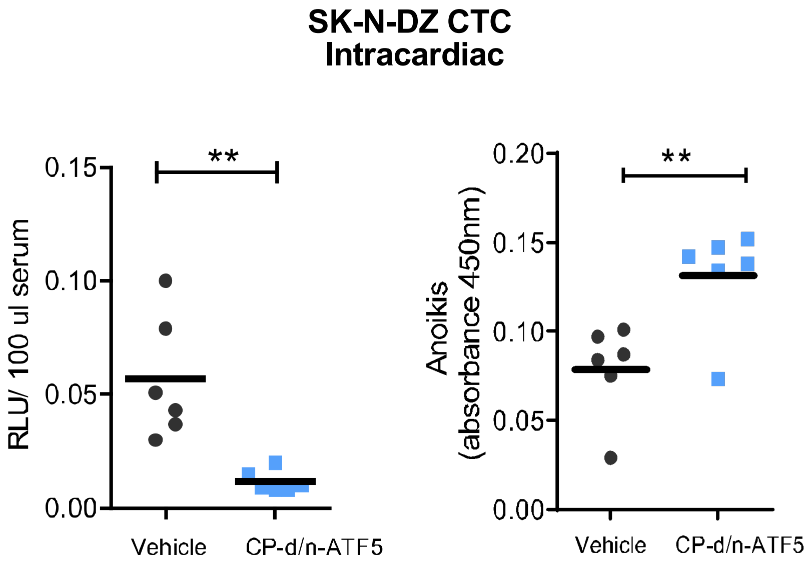

Supplementary Figure 18. CP-d/n-ATF5 treatment decreased viability and induced anoikis of SK-N-DZ CTCs. Quantification of SK-N-DZ CTCs measured by bioluminescence from blood (left), and quantification of anoikis in SK-N-DZ circulating cells (right) 12 hours after intracardiac injection and CP-d/n-ATF5 treatment, n=6. **, P<0.01
